# Phonon-assisted nonlinear optical processes in ultrashort-pulse pumped optical parametric amplifiers

**DOI:** 10.1038/srep23031

**Published:** 2016-03-15

**Authors:** Oleksandr Isaienko, István Robel

**Affiliations:** 1 Chemistry Division, Los Alamos National Laboratory, Los Alamos, NM 87545, US.

## Abstract

Optically active phonon modes in ferroelectrics such as potassium titanyl phosphate (KTP) and potassium titanyl arsenate (KTA) in the ~7–20 THz range play an important role in applications of these materials in Raman lasing and terahertz wave generation. Previous studies with picosecond pulse excitation demonstrated that the interaction of pump pulses with phonons can lead to efficient stimulated Raman scattering (SRS) accompanying optical parametric oscillation or amplification processes (OPO/OPA), and to efficient polariton-phonon scattering. In this work, we investigate the behavior of infrared OPAs employing KTP or KTA crystals when pumped with ~800-nm ultrashort pulses of duration comparable to the oscillation period of the optical phonons. We demonstrate that under conditions of coherent impulsive Raman excitation of the phonons, when the effective χ^(2)^ nonlinearity cannot be considered instantaneous, the parametrically amplified waves (most notably, signal) undergo significant spectral modulations leading to an overall redshift of the OPA output. The pump intensity dependence of the redshifted OPA output, the temporal evolution of the parametric gain, as well as the pump spectral modulations suggest the presence of coupling between the nonlinear optical polarizations P^NL^ of the impulsively excited phonons and those of parametrically amplified waves.

Nonlinear optical (NLO) frequency conversion techniques have become an indispensable tool in fundamental research and industrial applications to generate coherent laser emission away from the frequencies provided by laser gain media. Various NLO processes, such as optical parametric generation/amplification (OPG/OPA), optical parametric oscillation (OPO), harmonic generation etc., allow the generation of tunable laser pulses from the deep-UV to the far-infrared and terahertz (THz) spectral regions, with femtosecond to nanosecond pulse durations. The efficient implementation of these processes relies on the use of materials with high optical nonlinearities and appropriate phasematching conditions for various wave-mixing schemes.

One of the fundamental reasons behind the successful application of various NLO crystals–such as beta-barium borate (BBO), lithium borate, potassium titanyl phosphate (KTiOPO_4_, KTP) and many others–in frequency conversion schemes, is the instantaneous nature of the NLO response, most importantly the 2^nd^-order susceptibility *χ*^*(2)*^. Namely, electrons in these materials respond to the electric field oscillations of the interacting waves instantaneously, as long as the electronic susceptibilities *χ*^*(i)*^ are not coupled to any resonance in the NLO material. For this reason, frequency conversion in NLO crystals can be carried out practically without a spectral gap over the entire transmission range of each given material (with efficiency being limited only by the phasematching condition)^1^,^2^. On the other hand, the resonant modulation of optical nonlinearities has been actively employed, for example, in the spectroscopy of interfaces where the interfacial 2^nd^-order NLO response is non-zero as a consequence of inversion symmetry breaking^3^,^4^. The effective interfacial nonlinearity 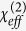 in this case is a coherent superposition of the nonresonant (instantaneous, electronic) 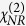 and the resonant (non-instantaneous) 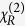 responses: 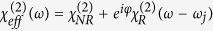, where *φ* is the phase offset (“lag”) between the nonresonant and resonant responses, and *ω*_*j*_ is the frequency of a certain NLO-active resonance mode. Taking advantage of this enhancement, various *χ*^*(2)*^-based schemes have been employed in spectroscopy of interfaces, in which either one of the exciting beams, or the generated (second-harmonic or sum-frequency) beam, is in resonance with the vibrational or electronic mode(s) at the interface. While the latter phenomenon has been employed in surface science for almost 30 years, the coupling of resonances in NLO crystals to the electronic polarizability–which typically is assumed to be negligible–as well as its implications for the efficiency of the frequency conversion devices, still need to be investigated in greater detail.

Recently, the topic of coherent interactions between electromagnetic radiation and the vibrational modes in the NLO crystals has been receiving a revived interest in light of the ongoing developments in terahertz parametric oscillators (TPOs)^5^,^6^,^7^,^8^,^9^,^10^,^11^,^12^ and Raman lasers^13^,^14^. In this regard, KTP and its isomorphs such as potassium titanyl arsenate (KTiOAsO_4_, KTA), RbTiOPO_4_ etc., represent a versatile group of NLO materials that possess relatively high 2^nd^-order nonlinear coefficients^15^, wide spectral transparency window (~400–4500 nm for KTP; ~350–5000 nm for KTA)^16^,^17^, and relatively high damage thresholds (reaching 100’s of GW/cm^2^ in femtosecond regime^16^). In addition, these materials exhibit characteristic Raman-, and at the same time, infrared- (IR-) active transverse-optical (TO) phonon modes in the ~7–20 THz range that correspond to the bending (δ_1_~7 THz; ~230 cm^−1^; ~28.5 meV) and stretching (δ_2_~20–21 THz; ~700 cm^−1^; ~85 meV) vibrations of the TiO_6_ octahedron structural unit in KTP/KTA^14^,^18^,^19^,^20^,^21^. With such a unique combination of properties, KTP and its isomorphs have found themselves in a wide variety of electro-optical applications. Many of these are driven mainly by the instantaneous off-resonant χ^(2)^ nonlinearities, for example, doubling of Nd-doped gain medium lasers^15^, OPO in the visible-to-near-infrared^14^, OPA of ultrashort pulses in the near- and mid-infrared^16^,^17^,^22^,^23^,^24^,^25^,^26^, and applications in χ^(2)^-cascading processes^27^. Moreover, one can also distinguish a group of NLO processes that involve the above-mentioned TO-phonon modes and that are inherently connected to the third-order optical nonlinearity χ^(3)^. These are represented primarily by: 1) the stimulated Raman scattering (SRS) and Raman lasing;^13^,^14^,^28^ and 2) the stimulated phonon-polariton scattering (SPS) and generation of laser beams in the terahertz (THz) frequency range^7^,^9^,^10^,^12^,^29^.

Previous investigations of KTP- and KTA-based parametric generators showed the possibility of SRS processes coexisting with the main parametric conversion^18^,^19^,^30^. Notably, the SRS of the parametrically amplified signal wave was shown to enhance when the idler wave was tuned into resonance with the second overtone band of the phosphate ion^18^ and, moreover, when the pump pulse duration was shortened to a few-picosecond durations^19^ thus approaching the dephasing times of the Raman-/IR-active phonons^31^. A distinct feature of these studies that utilize picosecond (relatively narrowband) pump pulses is that the Raman scattering occurs in cascading manner to the parametric processes^11^,^30^,^32^,^33^, i.e. the Stokes-shifted signal/pump spectra can be clearly distinguished from the major peaks corresponding to the OPO-generated signal and idler waves. In other words, the OPO is controlled by instantaneous χ^(2)^, while the SRS is controlled by the χ^(3)^ nonlinearity, and there is no direct modulation of one process by another.

In this work, we investigate the effects that arise within the OPA process in KTP and KTA crystals when TO phonons are impulsively excited by broadband, ultrashort (~50-fs) pulses whose duration is shorter or close to the oscillation period of the phonons, and shorter than the phonon decoherence time *τ*_*coher*_ . Under such conditions of impulsive excitation, we observe redshifts from the expected wavelength values in the signal pulse spectra (while the idler spectra remain practically unmodified) that correlate with the frequencies of the two most intense phonon modes δ_1_ and δ_2_ in the Raman spectra of KTP and KTA. The pump power dependence of the OPA output intensity can be modeled as being governed by an effective nonlinearity 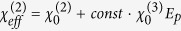, where 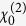 and 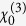 are intrinsic nonlinear susceptibilities, and *E*_*p*_ is the electric field amplitude of the pump wave. This observation, together with the dependence of the signal redshift on the pump pulse duration and our spectrally resolved pump depletion measurements, leads us to the conclusion that in the conditions of ultrashort (impulsive) pumping, the nonlinear optical polarization P^NL^ of the impulsively excited phonons becomes strongly coupled with the nonlinear polarizations of the parametrically-amplified waves, thus causing the observed redshifts in the OPA process. When accounting for this coupling, the effective χ^(2)^ nonlinearity that drives the OPA process cannot be considered instantaneous anymore, as opposed to the traditional theory of OPA action. This conclusion is supported by the temporal evolution of the parametric gain in KTP and KTA crystals. To our knowledge, such behavior of an OPA system where the resonant modes of the NLO crystal become coupled to the 2^nd^-order NLO process, has not been reported before. We discuss the implications of these findings for the possibility of the generation of ultrashort THz pulses in KTP and KTA crystals.

## Results and Analysis

### Initial observation of the redshift in the KTP and KTA OPAs

**Figure 1:**
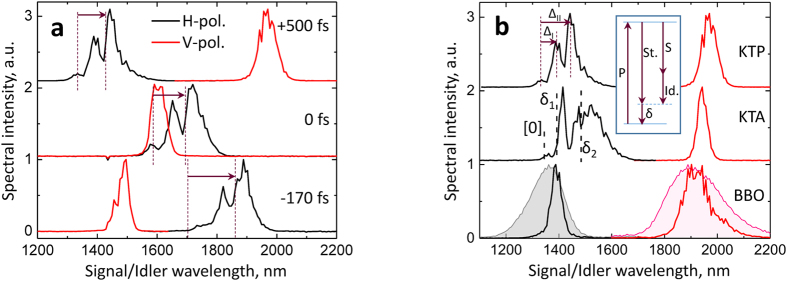
Initial observation of the signal/idler redshift in a KTP- and KTA-OPA, at the highest available pump pulse energies at the NLO crystal. (**a**) Representative pairs of signal-idler spectra from the KTP-OPA with the pump pulse compression optimized on the KTP crystal, with the corresponding polarizations. Before the NLO crystal, the seed beam is sent through a ~5-mm thick ZnSe plate for a more reliable tuning of the OPA (Methods). The corresponding seed-pump delays are shown. The arrows indicate the signal redshift Δ of the center-of-mass signal frequency from that expected based on the idler and pump positions. (**b**) Pairs of signal-idler spectra from the KTP-OPA (at +500-fs delay in a, shown again for clarity) and KTA-OPA, in comparison with that from a type-II BBO-OPA. The pump pulses are compressed on the NLO crystal in all cases. Black- (red-) line spectra correspond to horizontal (vertical) polarization of the detected waves. Δ_I_ and Δ_II_: characteristic frequency shifts in signal spectra of KTP- and KTA-OPA from the frequency expected from the idler position. [0], δ_1_ and δ_2_ (shown for KTA-OPA spectra): expected frequencies of the non-shifted (“intrinsic”) signal, and signal peaks shifted by the respective Raman modes, all based on the idler position, and 795-nm pump. Shaded: OPG spectra of a signal-idler pair from an unseeded BBO. Inset: energy diagram of interacting waves; P, pump; St., Stokes-shifted pump; S, signal; Id., idler; δ, phonon mode(s) of the crystal.

The OPA layout is based on the common design employing single-filament white-light continuum (WLC) as the seed, generated by focusing a few μJ of the 800-nm pulses into a 5-mm thick sapphire plate^1^ (see Methods for more details). Type-II phasematching allowed separating the signal and idler beams for individual spectrum measurements by using a calcite polarizer. The latter, in turn, allows distinguishing between the signal (“H-pol.” in Fig. 1) and the idler (“V-pol.” in Fig. 1) waves at, and even past, the degeneracy point of the signal seed tuning, achieved by the simultaneous adjustment of the seed-pump delay and the NLO crystal angle θ to optimize the total signal + idler power. Throughout the tuning curve of KTP- and KTA-OPA, the redshift Δ is strongly pronounced (mainly in signal spectra) and fluctuates around ~15 THz. Numerically, Δ is expressed as the frequency (energy) offset of the signal spectrum center-of-mass *ν*_*s,CM*_ from the expected difference frequency between the pump (*ν*_*p*_) and idler (*ν*_*i*_) peak positions: 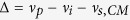 (horizontal arrows in Fig. 1a). While the signal spectra have a certain structure (which is analyzed in more detail below), the energy shifts calculated via the center-of-mass of spectra provide a good measure of the trends in the behavior of the OPA under various pumping conditions.

**Figure 2:**
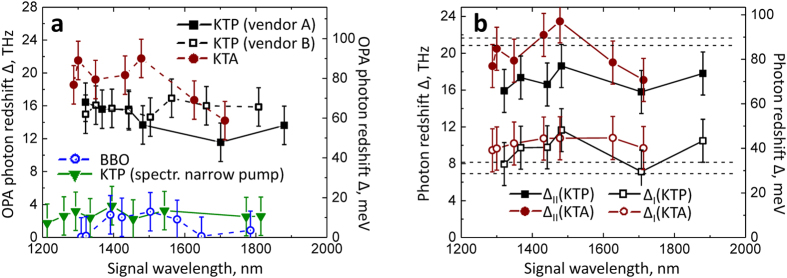
Redshift in KTP- and KTA-OPA as a function of the signal/idler wavelength, at the highest available pump pulse energies at the NLO crystal. (**a**) The photon energy redshift Δ measured over the tuning range of signal (e-polarized) in corresponding type-II OPAs. (**b**) Center-of-mass signal wavelength dependence of characteristic peak shifts Δ_I_ and Δ_II_ in signal spectra of KTP- and KTA-OPAs. Dashed horizontal lines represent the frequencies of the strongest modes in Raman spectra of KTP (8.06 and 20.8 THz) and KTA (7.0 and 21.8 THz). The vertical error bars correspond to the resolution of the InGaAs spectrometer (Supporting Information, Fig. S1).

The signal/idler spectra were measured in the same conditions of the pump compression on the NLO crystal, with the KTP, KTA, as well as with a BBO crystal. Figure 1b shows three pairs of signal/idler spectra from these OPAs, at close idler peak positions, demonstrating the persistence of the redshift in the KTA-OPA and its disappearance in BBO-OPA. The redshift energy appeared to be largely independent of the signal or idler wavelength (Fig. 2a) for the entire spectral region of interest. The latter suggested that the OPA process is coupling to some nonlinearities that are not sensitive to phasematching conditions (e.g. Raman-excitation processes). The spectral offsets of the two main redshifted components in the signal spectra from KTP and KTA OPAs (Δ_I_ and Δ_II_, as introduced in Fig. 1b) appeared to correlate with the frequencies of the δ_1_ and δ_2_ phonon modes in KTP and KTA (Fig. 2b). In general, however, the apparent peak positions are slightly different from the expected ones based on δ_1_ and δ_2_ (see KTA-OPA spectra in Fig. 2b), which indicated a possibility of complicated interference between the modes that leads to the observed structure of signal spectral shape (a more detailed analysis of signal spectra is given in the next sub-section). Lastly, we note also that the bandwidth of the signal and idler pulses from OPG in BBO was at least 2 times wider than that of the seeded BBO-OPA output (Fig. 1b). This demonstrates that the use of the temporal stretcher of white-light continuum pulses (see Methods) indeed improved the selection of the spectral components from the broadband white-light seed pulses by the ultrashort pump. Besides, no redshifts were observed in the unseeded OPG spectra from BBO itself, which further indicated that the redshift in the output from of the KTP/KTA is an actual physical phenomenon, and not an artifact introduced by the spectroscopic measurement.

To test the role of impulsively excited phonons in causing the OPA redshift, we investigate the dependence of Δ on the pump pulse bandwidth, which in turn determines the transform limit of its time duration. When the pump spectrum was narrowed by inserting a ~5-mm wide slit into the spatially dispersed beam in the amplifier compressor (to ~12–15-nm bandwidth from the Δλ ≈ 25 nm original value), the redshift Δ was significantly suppressed (Fig. 2a, green triangles). Moreover, by gradually adjusting the width of the slit, we were able to “tune” the redshift in the conditions of close pump pulse energies at the KTP crystal (Supporting Information, Fig. S2). While the phonon modes have well-defined frequencies, the contribution of the redshifted modes in the signal spectra varies with the pump bandwidth, thus leading to a gradual change of the overall redshift Δ. These results indicate that the proximity of the pump pulsewidth to the characteristic oscillation periods of resonant modes in KTP (as well as its isomorphs) is critical for the onset of redshift in the KTP-type OPAs. In order to further investigate the physical origin of the redshift, we have: (i) studied the pump intensity dependence of the parametrically amplified signal/idler pulse spectra, (ii) measured the spectral depletion of the pump pulses and (iii) investigated the temporal evolution of the parametric gain.

### Pump pulse intensity dependence of the redshift in KTP and KTA OPAs. Coupling between χ^(2)^ and χ^(3)^ nonlinearities

**Figure 3:**
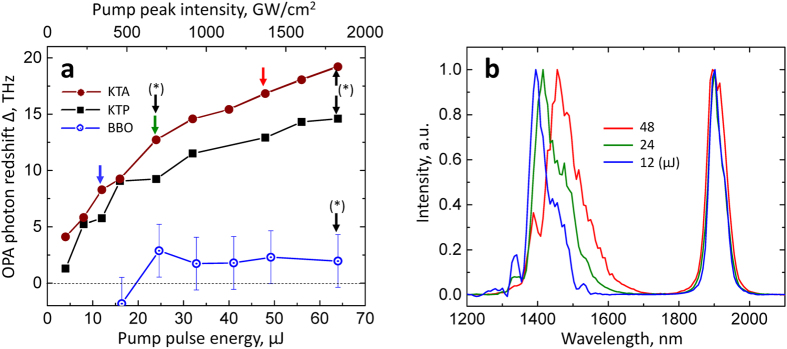
(**a**) Pump-intensity dependences of the OPA photon redshift Δ in corresponding nonlinear optical crystals, measured in the same conditions (in all cases, the pulse compression is optimized on the crystal). (**b**) Signal/idler spectra in KTA-OPA at selected pump energies. Spectrum colors correspond to the color-coded arrows in (**a**) indicating the data points at the respective pump pulse energies. The asterisk-labeled data points in (**a**) correspond to the pump pulse energy values at which the pump spectral depletion was measured in respective NLO materials.

The dependence of the signal/idler spectra on the pump pulse energy, and consequently, on the peak intensity, was investigated at a few signal/idler pairs of close wavelengths in KTP-, KTA-, as well as in BBO-OPA. In the initial step, we analyze the pump power dependence of the overall redshift Δ of the signal pulses in the three materials (Fig. 3). In one of the horizontal axes for this dependence, we plot the pump pulse peak intensity estimated for the beam spot diameter ~300 μm (from knife-edge measurements) and the ~50-fs pulsewidth (based on previous autocorrelation measurements of the pump laser, as well as pump-seed crosscorrelation, see below). The highest used pulse energies correspond to peak intensities that are comparable or above the damage threshold of KTP estimated from the data at ~120-fs pulsewidth^16^ and the 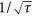 scaling law. Nevertheless, we did not observe any drastic change in the outputs of KTP/KTA OPAs over several hours after optimization at various signal/idler wavelengths (although, the optical damaging may be expected to accumulate on longer timescales). A factor that might have prevented the damage in these OPAs was the conversion of the pump pulses during the seeded-OPA process: we observed an onset of bright continuum emission from KTP/KTA crystals when the seed beam was blocked (see also the Discussion section).

While for BBO-OPA the red-shift values stay practically negligible (if compared to the instrument’s resolving ability) over the entire range of available pump pulse energies, the value of Δ apparently decreases as the pump pulse energy is decreased at the KTP and KTA crystals. Interestingly, a considerable redshift is still persistent even for the pump pulse intensities that are below the onset of white-light continuum generation in the NLO crystals (occurring at ~45–50 μJ pump energy, corresponding to *I*~1250 GW/cm^2^; Fig. 3a), thus indicating that it is not likely to be caused by self-phase modulation as a major mechanism for its appearance. Another important observation is that the idler spectra remain largely unchanged, exhibiting in some cases a shoulder in the relatively narrowband peak (FWHM bandwidths <6 THz).

The straightforward and somewhat simplified analysis presented above already demonstrates that the OPA process in KTP and KTA must be undergoing certain pump-intensity dependent coupling to another NLO process. The shifts present in the signal spectra, as well as their insensitivity to phasematching conditions, strongly suggest that this NLO process must be the impulsive stimulated Raman excitation of the TO phonons in KTP and KTA. Phenomenologically, the impulsive excitation creates a macroscopic nonlinear polarization 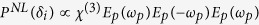 at each respective phonon frequency δ_i_^34^. In the case of KTP- and KTA-OPA, the pump pulse duration is shorter than the phonon dephasing time, and thus the interaction between the second-order nonlinearity that drives the OPA process and the pump/signal/idler waves may not be considered strictly as instantaneous (as it is traditionally accepted in the theory of optical parametric amplification). In these conditions, the second- and third-order optical nonlinearities may become coupled, e.g. ***χ***^***(2)***^_***eff***_ = ***χ***^***(2)***^_***ijk***_ + *const*.***χ***^***(3)***^_***ijkl***_ *:**E***_*l*_ thus leading to the appearance of nonlinear polarizations at frequencies *(ω*_*j*_ − *δ)*, where *j* = pump, signal, or idler. In the spectral domain, such interaction can be understood as the OPA process utilizing the Stokes-scattered pump wave (inset in Fig. 1b). This coupling mechanism is also supported by the time-domain description (see below).

Following above considerations and at high enough electric fields of the pump wave, the effective second-order nonlinearity may be modified as:


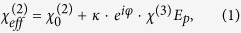


where κ is a coupling coefficient (takes into account, e.g. the orientations of beam polarizations and the crystal axes), *e*^*iφ*^ is the phase delay factor between responses of the 2^nd^- and 3^rd^-order nonlinearities. Note that Eqn. (1) considers the modulation by the pump electric field as the most intense one. The latter suggestion appears to be a realistic assumption in our experiments. The third-order nonlinear susceptibility of KTP and KTA crystals is ~10^−21^ m^2^/V^2^ 35,36, thus at the estimated pump peak intensities (exceeding ~1000 GW/cm^2^, corresponding to electric field amplitudes on the order of ~2.10^9^ V/m), the absolute values of 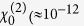
m/V) and 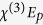 become comparable.

Using the pump power dependence of various modes within the signal spectra, it is possible to verify whether the coupling mechanism described by Eqn. 1 may be responsible for the observed redshifts in KTP- and KTA-OPAs. By applying the slowly-varying amplitude approximation, and neglecting the pump depletion, one obtains the following formula for the amplification factor of the signal seed from the intensity I_s_(0) at the entrance of the NLO crystal to the intensity after interaction length L, I_s_(L), in a single-pass OPA at zero phase-mismatch^1^:


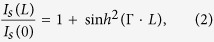


where


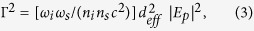


ω_i_ and n_i_ (ω_s_ and n_s_) are the radial frequency and index of refraction of idler (signal), *c* is the speed of light, and 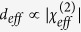 is the effective nonlinear optical coefficient. Thus, the intensity (power) of frequency components in the signal spectra that originate from the phonon-induced interactions between the 2^nd^- and 3^rd^-order optical nonlinearities are expected to follow the expression:


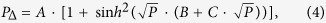


where *P* is the pump power; 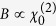, 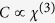 and *A* are fit parameters (ignoring the imaginary part of the phase factor in Eqn. (1)). At the same time, the intensity (power) of frequency components in the signal spectra that originate from the direct amplification of the seed photons P_0_ at a given phase-matched wavelength (“intrinsic signal” modes) are expected to follow Eqn. 4 as well, however with C≡0. Alternatively, one can fit the power dependence of the intensities of the redshifted and “intrinsic” signal modes by a modified form of Eqn. 4:


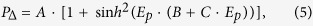


remembering that the electric field amplitude and the peak intensity *I* are connected by the expression 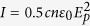, where n(KTP)≈1.8, ε_0_ = 8.854.10^−12^ C/(V.m), and *c* is the speed of light in vacuum.

**Figure 4:**
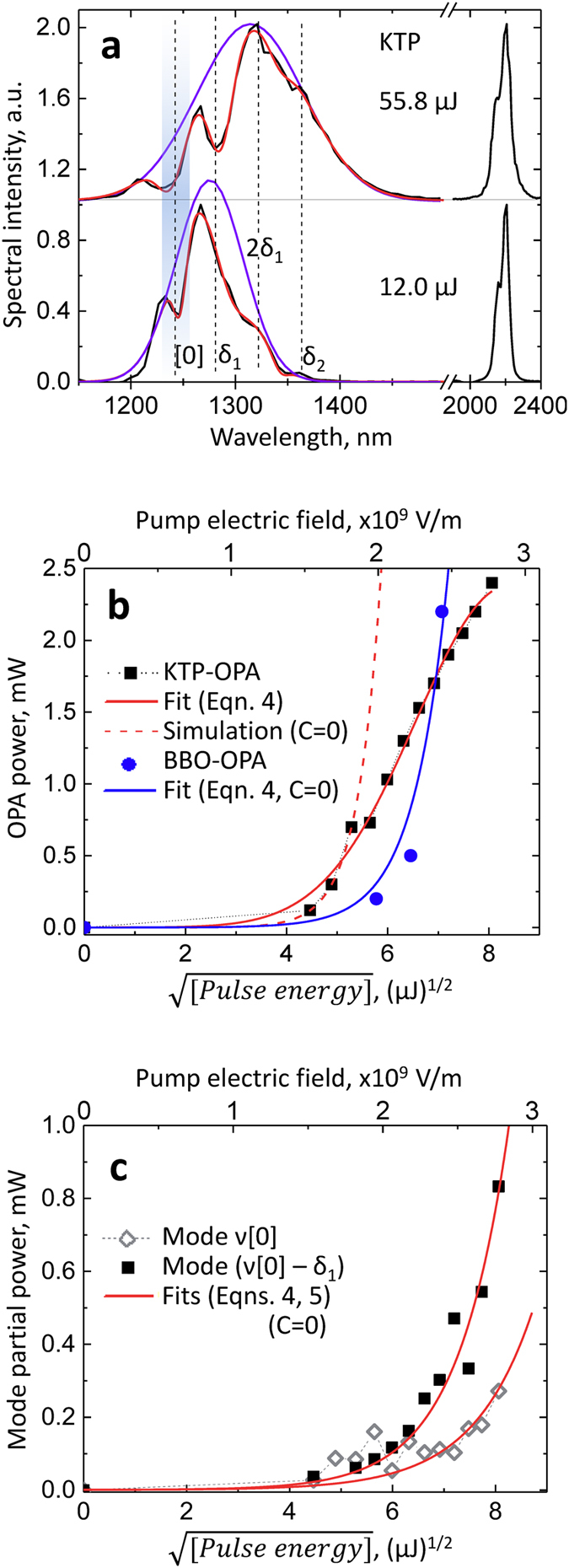
Analysis of the pump pulse intensity dependence of the signal spectral shape in KTP and KTA-OPAs. (**a**) Signal/idler spectra at two respective pump pulse energies in KTP-OPA. Shaded-dashed vertical line ([0]): “intrinsic” signal peak position (~1245 nm) based on the idler spectrum (~2200 nm) and 795-nm pump. The width of the shaded band indicates the spectral resolution (upper value). Vertical dashed lines δ_1_, 2δ_1_, δ_2_: positions of signal peaks shifted by the respective frequencies. Red lines: four-Gaussian fits of the signal spectra; the envelope spectrum (violet) has the positive amplitude, while the other three modes have negative amplitude (see Supporting information for the compilation of fit results, Table S2). (**b**) Pump-pulse energy (electric field, for top x-axis) dependences of the total KTP- and BBO-OPA power outputs, together with the fits to Eqns. (4) and (5); see Table 1 for fit results. The red dashed line is a simulation with C=0, and adjusted parameter A. (**c**) Pump pulse intensity dependence of the partial power of modes at ν[0] and ν[0]–δ_1_ together with the fits (red lines) to Eqns. (4) and (5).

The considerations above allow us to apply a more quantitative approach to the analysis of pump power dependence of KTP- (KTA-) OPA output. First, we note that while the idler spectra remain practically unchanged, the signal undergoes a pronounced variation in the spectral shape with the pump intensity (Figs 3a and  4a). This may be explained by the fact that the signal wave has a non-zero polarization component parallel to the z-axis of KTP (or KTA) crystal as pointed out earlier for polariton scattering in KTP^9^. Alternatively, the normal orientation of the signal polarization to the pump wave may lead to the strongest coupling of the signal to the TO phonons. Next, the spectral shape of the signal pulses is rather complicated. Even at the lowest pump pulse energies at which the OPA spectra could be measured (and at which the OPA-Raman coupling is expected to be minimal), the signal spectra have certain modulations and do not appear as a single peak at the expected frequency ([0] in Fig. 4a). Moreover, instead of a peak at the “intrinsic“ signal frequency, in many cases there is a pronounced “dip”; similar modulations are observed at frequencies at which we would expect the signal peaks to appear due to shifts on modes δ_1_ and δ_2_. These observations indicate that the homodyne-measured signal spectra (proportional to 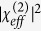) are the result of complex interference between χ_0_^(2)^ and spectrally-dependent χ^(3)^(δ) (see Eqn. 1). This phenomenon is similar to the phase-dependent spectral shape variations in homodyne-measured spectra 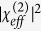 of the interfacial water molecular vibrations when the resonant (vibrational) and nonresonant (electronic) contributions into 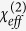 are of comparable magnitudes^37^,^38^. The proper decomposition of signal spectra in this case would require involvement of the spectral shapes of the nonlinear susceptibilities, as well as the knowledge of the relative phase φ of Eqn. 1 for each mode which, in turn, would potentially require certain heterodyne (phase-sensitive) measurements.[Table t1]

To analyze the pump intensity dependences of the spectral variations in KTP- and KTA-OPAs, we first apply Eqns. 4 and 5 to fit the total OPA output power values. We do this based on the notion that the signal spectra have, in general, a large contribution from the redshifted components in broad ranges of the pump intensities (Supporting Information, Tables S1, S4). This allows us to approximate the power of the signal beam as being proportional to the intensity of some “averaged” redshifted mode (thus neglecting the contribution from the “intrinsic” signal), assuming that the coupling strength for both δ_1_ and δ_2_ phonon modes is the same. Figure 4b demonstrates that this approach describes well the pump power dependence of the total OPA output (see Supporting Information for additional sets of data and fits; Figs S3–S5; Tables S1–S6). Equation 4 with C=0 cannot describe the pump power dependence for KTP-/KTA-OPA (dashed line in Fig. 4b) indicating that one does need to include a term proportional to 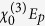. Moreover, the signs of the coefficients B and C are opposite, further supporting the above suggestion that the second- and third-order nonlinearities may be destructively interfering at certain wavelengths, i.e. φ ≈ π in Eqn. (1). Both 2^nd^ and 3^rd^ order nonlinear susceptibilities were shown to have the same sign in KTP^36^. We also note that the B coefficients for KTP and BBO fit results (Eqn. 5) are close to the effective nonlinearities of the respective processes^39^.

Next, we attempt to extract the amplitudes of the spectral components that would correspond to the “intrinsic” signal peak(s). We treat each signal spectrum as an envelope Gaussian function (violet lines in Fig. 4a) which is modulated by Gaussian-shaped modes of opposite-sign (negative) amplitudes. Typically, there are three negative peaks (Fig. 4a): at the “intrinsic” signal position ([0]), at the signal frequency shifted by δ_1_ (mode ν[0]–δ_1_), and at the signal frequency shifted by a value between 2δ_1_ and δ_2_. This fitting provides us with the relative amplitudes A_j_ and bandwidths Δλ_j_ for each apparent spectral mode, allowing us to calculate the partial power proportional to each mode 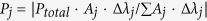, where *P*_*total*_ is the total signal + idler power measured at each given pump pulse energy (the change of relative signal/idler photon energies with pump power is only a few percent; see Supporting Information for tabulated results of analysis).

Figure 4c shows that the partial powers of modes at ν[0] and ν[0]–δ_1_ in the signal spectra can be fitted with Eqn. 4 when C=0, thus suggesting that they correspond to the OPA of signal photons unaffected by the phonon interactions (out of phase with the effective nonlinearity). This notion is supported by the negative amplitudes of these modes which destructively interfere with the envelope spectra (the latter most likely correspond to the effective parametric gain spectral profile, see the Discussion session). Similar results were obtained for KTP- and KTA-OPA at other signal/idler wavelengths (Supporting Information). The occurrence of two peaks (and not just one) behaving as the “intrinsic” signal mode correlates with the double-peak structure of scattered pump spectra (see next sub-section). Identifying the exact nature of the third peak (between ν[0]–2δ_2_ and ν[0]–δ_2_) would require further studies and analysis as this mode always appeared on the spectrum shoulder and thus its position could vary widely between spectra. While our analysis captures the essential experimental trends, we note that a more rigorous approach would take into account that: (i) in general, *χ*^*(2)*^_*eff*_ must be analyzed as a complex function; (ii) variation of the relative phases and amplitudes may affect the spectral shapes of modes imposed on the envelope spectrum (e.g. they may appear as “derivative-like” features)^37^.

### Spectral modulation and depletion of the pump pulses

Previous studies of SRS and stimulated polariton scattering in KTP and its periodically-poled forms^18^,^19^,^29^,^30^ clearly indicated spectral shifts in the scattered pump pulses which helped derive the modes that are responsible for the scatter of each given Stokes beam. The unambiguous determination of the values of Stokes shifts in the scattered beams in these studies was possible due to narrow spectral widths of the picosecond pump pulses compared to the frequencies of the phonons excited in the NLO crystals.

**Figure 5:**
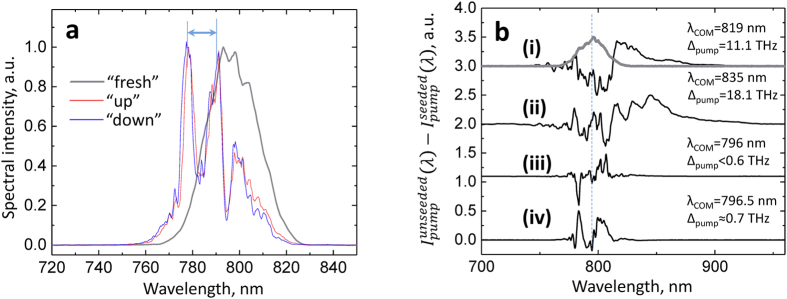
Spectral modulation of pump pulses. (**a**) Spectra of polariton-scattered pump photons detected after KTA in both directions (“up” and “down”) at ~1.8° external angle in vertical plane, measured in X(Z, Z + Y)X configuration, together with the pump spectrum before the KTA crystal (“fresh”). The double arrow indicates ~210–220 cm^−1^ splitting (≈δ_1_). (**b**) Pump difference spectra (unseeded minus seeded OPA) showing the spectral depletion of pump pulses. (i) KTP-OPA, high pump pulse energy (>60 μJ; OPA redshift Δ ≈ 12–14 THz); (ii) KTA-OPA, high pump pulse energy (>60 μJ; Δ ≈ 16–18 THz); (iii) KTA-OPA, low pump pulse energy (<25 μJ; Δ < 8 THz); (iv) BBO-OPA, high pump pulse energy (>60 μJ; Δ < 4 THz). Pump spectrum at the NLO crystal is shown as well in (i), with λ_0_ = 795 nm. Center-of-mass (COM) wavelengths (λ_COM_) and the shift due to depletion (Δ_pump_) are displayed in each case, together with the redshift in the OPA Δ (calculated from 795-nm).

In our case, the pump pulses are ultrashort (≤50 fs, see below) and thus can undergo significant spectral modulations when interacting with the impulsive Raman-/IR-active modes in KTP and KTA even at low intensities^40^. In fact, we observe the scattering of ~800-nm pulses on phonon-polaritons in KTP and KTA at small angles (~1° internal angle along the Y-axis). The spectra of polariton-scattered pulses are broadband as well, and display two main modes separated by ≈δ_1_ splitting (Fig. 5a). A likely reason of the additional blue shift in the scattered spectra is that the pump pulses act as an out-of-phase probe with the coherently excited phonons^40^.

In order to circumvent the complications imposed by the broadband nature of the pump pulses, we measured the spectral depletion of the pump pulses by acquiring the pump spectra after passing through the OPA crystal, and by subtracting the pump spectra after seeded OPA 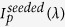 from the unseeded-OPA pump spectra 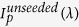 (Fig. 5b). This procedure was chosen as a means to observe what spectral components within the broadband pump participate in the process of energy exchange with signal and idler waves during the OPA. The pump spectral depletion measurement was performed in the three NLO materials under the same conditions of high pump pulse energy (Fig. 5b,i,ii,iv). This procedure accompanied the measurements of the redshift in the OPA output, as shown on the corresponding data points marked with asterisk in Fig. 3a. We also measured the pump spectral shift in KTP- and KTA-OPA at low pump pulse energies (result for KTA-OPA is shown in Fig. 5b,iii). In order to quantify the spectrum shift of the pump pulses from the center wavelength, the center-of-mass of the modulus of 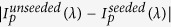 was calculated for each difference spectrum in order to account for the presence of negative values.

While for BBO-OPA the overall spectral shift of the pump pulses is less than 0.5 THz, below the resolution of our InGaAs spectrometer, the spectral shifts in KTP- and KTA-OPAs are significant (>10THz) and correlate with pronounced redshifted components in the amplified signal spectra. In fact, a closer examination of difference spectra in Fig. 5b,i,ii shows that there is a depletion of pump pulse components that are redshifted by >24 THz (~820 cm^−1^) from the spectrum peak. We also notice that in some instances it is possible to observe oscillations in the pump difference spectra, with a period close to frequencies of the TO phonon modes (e.g. ~6.9-THz oscillations in Fig. 5b,ii). Furthermore, for low pump pulse energies, the pump spectral shift becomes strongly suppressed (Fig. 5b,iii) which also correlates with the suppression of the signal redshift (Fig. 3) under the same pumping conditions.

In addition to correlation between the overall shifts in the pump depletion spectra and the redshifts in the OPA process, these results are important as they demonstrate that the redshifts in KTP- and KTA-OPAs cannot be due to such effects as participation of only one portion of the pump spectrum in the OPA process, e.g. due to some dispersion effects imposed on the pump pulses before they arrive at the NLO crystal. If the latter were true, it would also occur in BBO-OPA, however, we do not observe any considerable redshifts either in the OPA output or in the pump depletion spectra in BBO OPA (Fig. 5b,iv) at any pumping conditions.

### Temporal evolution of the parametric gain. Phasematching considerations

**Figure 6:**
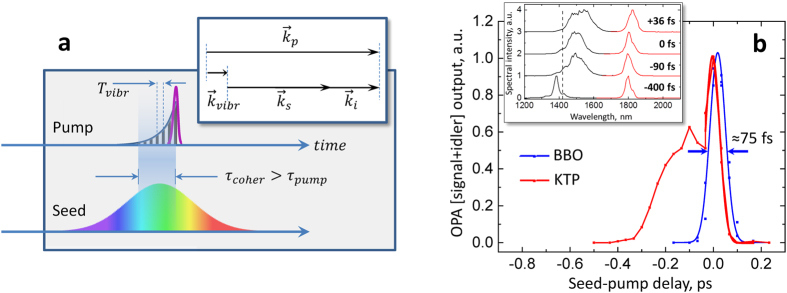
Time-domain description of coupling between the 2^nd^-order nonlinearity and the Raman-/IR-active intrinsic phonon modes of the NLO crystal. (**a**) An ultrashort pump pulse (purple) causes impulsive excitation of the lattice vibrations (vertically-hashed) which trail behind the pump pulse and decay over the period of time *τ*_*coher*_. The seed photons interact with the excited lattice over the *τ*_*coher*_ time period, thus the effective nonlinearity χ^(2)^ becomes time-dependent (non-instantaneous). Inset: Phase-matching scheme; the wave-vector of the transient grating that appears due to the coherent lattice vibrations (***k***_*vibr*_) becomes involved in the parametric process, similar to the phase-matching in periodically-poled crystals. (**b**) Total OPA output power measured for KTP and BBO crystals as a function of the seed-pump interpulse delay. The front (trailing) edge of the pump pulse is at the positive (negative) delay values. For these measurements, the ZnSe plate was removed from the white-light beam path, and the crystals were tuned to select the seed wavelength components close to ~1500 nm where the group-velocity dispersion imposed on the white-light continuum is minimal. The FWHM = 75 fs is indicated for the pump-seed cross-correlation in BBO; the FWHM for the instantaneous part of cross-correlation in KTP is ~70-fs, corresponding to ~49.6-fs pump pulsewidth as the upper-value estimate. Inset: pairs of signal (black) and idler (red) spectra from KTP-OPA at corresponding seed-pump delays. Dashed vertical line indicates the expected spectral position of the “intrinsic” signal peak based on 795-nm pump and 1800-nm idler wavelengths. The slight blue-shift of the signal spectrum from the expected position for the ≈–400-fs delay is explained by the OPA process being driven by the higher-frequency spectral components in the pump pulses after undergoing positive group-delay dispersion in KTP.

So far, we have discussed the coupling between the χ^(2)^ and χ^(3)^ nonlinearities in KTP and KTA as it is studied in the frequency domain. An apparent consequence of such coupling of the parametric process to the intrinsic, Raman-/IR-active resonances is the appearance of redshifted components in the signal spectra. However, it is also imperative to discuss such coupling in the *time domain*, especially because only such consideration can truly demonstrate how the 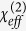 becomes non-instantaneous. The characteristic difference of our investigation from the previous studies of KTP- and KTA-OPAs is that the pump pulses possess the bandwidths that can lead to impulsive excitation of the TO phonons and that are ~4-5x shorter than the phonon decoherence (dephasing) lifetime^31^. The combination of the latter two factors leads to the creation of a transient grating of coherent lattice vibrations that trails behind the pump pulse (in space and time), with a period corresponding to the natural oscillations of the intrinsic vibrations and decays over the decoherence time *τ*_*coher*_ (Fig. 6a). It is within this period of time *τ*_*coher*_ that the second-order susceptibility of the NLO crystal becomes modified according to Eqn. 1 (thus leading to the appearance of additional frequencies in the signal spectra).

Such description of the OPA process in the presence of impulsively-excited vibrations in the NLO material also allows to consider the phase-matching condition (Fig. 6a, inset). Essentially, the NLO crystal becomes periodically-poled–over the period of time *τ*_*coher*_–with the poling period equal to the effective phonon wavelength which is determined as the distance traveled by the pump pulse during one oscillation period Λ = *cT*_*vibr*_*/n*, where n is the refractive index of KTP (KTA) at the pump wavelength^40^ (Fig. 6a). Such transient lattice grating has the wave vector 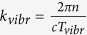, which modifies the phasematching condition for the OPA process: 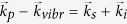, similar to that in periodically-poled crystals^26^,^41^.

In order to confirm the time-domain description of the coupling between OPA and the intrinsic phonon modes, we measured the OPA output power as a function of the pump-seed inter-pulse delay (Fig. 6b). In BBO-OPA, such cross-correlation is essentially pump pulsewidth-limited (demonstrating instantaneous χ^(2)^ in this material). On the other hand, in KTP-OPA there is a delayed response that follows the initial instantaneous signal; the delay time (~150–200 fs) is characteristic of the decoherence time of TO phonons in KTP^31^. The temporal evolution of the parametric gain correlates with the spectral modulation of the signal pulses (Fig. 6b, inset): the signal spectra are redshifted already at the front-edge of the pump pulse, and this modulation persists until the coherent phonon vibrations decay completely. In addition, beat components may be noticed in the time-dependent traces, however their proper analysis requires the acquisition of such cross-correlations at a higher temporal resolution. KTA demonstrated very similar behavior (Supporting Information, Fig. S6). These measurements in the time domain are consistent with the frequency-domain studies and further support the notion that in materials with resonances whose response is slower than the pump pulsewidth, it is possible to observe various effects due to the coupling between the instantaneous and resonant responses.

## Discussion

It is worthwhile to point out a few additional observations based on the results described above. The disappearance of the redshift in the OPA output, when replacing KTP or KTA with a BBO crystal, is consistent with the fact that the third-order nonlinearity in BBO is much weaker than in KTP or KTA^35^,^36^,^42^,^43^. Moreover, the fits of the KTP-OPA output pump-power dependences (Fig. 4b) to Eqn. (5) yield the ratio 
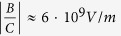
which is of the same order of magnitude as 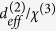 in KTP.

The envelope functions that are derived from the signal spectral decomposition (Fig. 4a; Tables S1 and S4, Supporting Information) most likely correspond to the spectral profiles of the parametric gain in KTP/KTA at various pump intensities. Their FWHM bandwidths vary from ~60 to ~90 nm corresponding to ~16–25 THz at ~1300–1400 nm. The broad bandwidth of signal spectra at early seed-pump delays (inset in Fig. 6b; ≈+30–40 fs delay) indicates that both phonon modes δ_1_ and δ_2_ of the crystal lattice are excited already at the front edge of the pump pulse thus dictating the overall spectral profile of the parametric gain. The bandwidths of the envelope modes are comparable to those of the OPG spectra from the unseeded BBO (FWHM~150 nm at ~1370 nm, or 24 THz; Fig. 1b, shaded). The difference is, however, that we could never clearly observe the OPG spectra from unseeded KTP or KTA when pumped in the same conditions as the BBO crystal. This could be a consequence of the scattering of OPG-photons on the excited phonon vibrations, thus manifesting the true parametric fluorescence, similar to the spontaneous band-edge fluorescence spreading into the 4π solid angle. However, as soon as the seed photons arrive, the parametrically-amplified photons get directed to maintain the phasematching condition.

The estimated value of the damage threshold in KTA and KTP (~600–800 GW/cm^2^) at ~50-fs pump pulsewidth based on the literature data^16^ is below the intensity at which we observed the continuum onset from unseeded NLO crystals suggesting that the actual damage threshold may be even higher. An additional factor that may be preventing the optical damage is the partial and reversible disordering of the crystalline structure due to the impulsive excitation of the phonon modes, following the discussion by Pasiskevicius *et al.* in an earlier study of SRS in KTP with picosecond pump^19^. As our data show (Fig. 3a), the effect of impulsive stimulated Raman scattering (ISRS) of phonon modes will always be present in OPAs based on either KTP or KTA with ≈50-fs pump, since ISRS is thresholdless. However the extent of the effect will be dependent on the strength of the χ^(2)^–χ^(3)^ coupling which, in turn, is dictated mostly by the pump intensity. In this respect, additional investigations would be needed on the optical damage in titanyl crystals when pumped in the ultrashort-pulse regime. The latter knowledge would help determine the extent of the observed phenomena within the ranges of the pump intensities relevant to the operation of common-design OPAs relying on titanyl materials.

Another consequence of the χ^(2)^–χ^(3)^ coupling is that the OPA output from KTP and KTA crystals is less than the corresponding value expected based solely on the χ^(2)^-nonlinearity. Our analysis of signal spectra shows that the partial power in the envelope spectral mode can reach ~4 mW (Supporting Information, panel b in Fig. S3 and S5), *vs* the measured ~2–2.5 mW. The expected values are close to the power outputs from the BBO-OPA measured in the same conditions. Thus, the photon-phonon coupling in KTP and KTA crystals might explain why these materials typically provide less amplification even though their nonlinear coefficients *d*^*(2)*^_*eff*_ are close to or exceeding that of BBO. On the other hand, the non-instantaneous response of *χ*^*(2)*^_*eff*_ may be beneficial for the operation of optical parametric chirped pulse amplifiers (OPCPA) relying on KTP or KTA^17^ as it relaxes the stringent conditions for the temporal synchronization of the seed and pump pulses^2^.

The similar behavior of KTA and KTP in OPA suggests that we should be able to expect terahertz pulse generation from the KTA material as well. KTP has been recently shown to produce terahertz pulses from TPOs in the nanosecond regime^7^ and to exhibit intense stimulated phonon-polariton scattering when pumped by picosecond pulses^9^,^19^,^29^. However, our results indicate also that KTP, and moreover KTA, are expected to produce ultrashort, broadband THz pulses when pumped in pulse-front tilted arrangement, similar to lithium niobate^44^. The indices of refraction for KTP in the vicinity of the optically-active phonon modes (e.g. n ≈ 3.3–3.9 at ~2 THz^12^; n ≈ 4.1 at 5.9 THz^9^) suggest that it is possible to phase-match the pump and Stokes components of 800-nm pulses (at ~10-THz separation, Fig. 5) with a phonon-polariton wave of ~3–5 THz frequency in noncollinear geometry (i.e. ~1–2° internal pump-Stokes angle). In order to take advantage of these effects for practical THz generation, the intended KTP (or KTA) crystal will need a proper geometry to accommodate small total-internal reflection angles of THz waves^7^.

In conclusion, we have investigated the effects of pronounced spectral modulations (mainly a red-shift of the signal beam) in the output of OPAs based on KTP and KTA NLO crystals imposed by ultrashort pump pulses of ~50-fs or shorter durations. This behavior is explained by the impulsive excitation of the Raman- and IR- active phonon modes in these materials, with the key realization that the 2^nd^-order nonlinear optical interactions cannot be considered as instantaneous anymore because the decoherence times of the phonons are much longer than the pump pulsewidths. The data suggest that the OPA process in these conditions becomes strongly coupled with the phonon nonlinear polarization and this coupling is pump-intensity dependent. To our knowledge, this is the first demonstration of the OPA process that gets affected by the resonances in the NLO crystals. It becomes of fundamental interest to investigate the possibility of similar processes in other NLO materials, especially those that possess slowly-decaying Raman-/IR- active phonon modes in the lattice vibrational spectra that can couple to the electromagnetic radiation.

## Methods

The front end laser source was a Ti:sapphire regenerative amplifier delivering 1 mJ pulses centered at 800 nm at 1 kHz repetition rate, with a Δλ~28-nm bandwidth supporting <40-fs pulsewidth (Spitfire, Spectra Physics). The total energy of pump pulses devoted for the OPA was ~100 μJ (selected with a beamsplitter). The central wavelength shifted to 795 nm at the front side of the NLO crystal due to the reflectivity spectra of the routing mirrors. The OPA layout was based on the common design employing single-filament white-light continuum (WLC) as the seed, generated by focusing 1–2 μJ of the 800-nm pulses into a 5-mm thick sapphire plate^1^. The WLC spectrum (Supporting Information, Fig. S7) in the near-infrared had the exponential dependence of the spectral intensity on the wavelength typically observed for the Stokes side of the continua^45^. The KTP and KTA NLO crystals employed in this study had the following parameters: cut for type-II phasematching in XZ plane (φ = 0°; θ = 42^o^ for KTP and θ = 45° for KTA); thickness ~3 mm and aperture 5 × 6 mm. In both NLO materials, the phasematching configuration was the following: the seed beam, serving as the signal in the ~1100–1600 nm range, was extraordinary- (e−) polarized (in XZ-plane; horizontal with respect to the laser table), while the pump and idler waves were ordinary- (o−) polarized (parallel to the Y-axis; vertical with respect to the laser table)^17^,^24^. The pulse energy of the pump at the NLO crystal was adjusted with a variable neutral density (VND) filter. The polarization of the pump pulses was rotated to vertical with a half-wave plate to fulfill the phasematching condition for the OPA process. The BBO crystal was 4-mm thick; cut for type-II phasematching, θ ~ 28°; e-pump, o-signal, e-idler; the crystal was rotated around a horizontal axis to maintain the same polarizations of signal and idler waves, as well as the same pump-seed delays, as in KTP- and KTA-OPAs. The tuning of signal/idler output was achieved by adjusting the phasematching angle theta (rotation of crystals around vertical axis; around the horizontal axis for BBO). In all measurements, the pump pulse compression was optimized on the NLO crystal, by either maximizing the infrared portion of the parasitic white-light continuum generation from the unseeded KTP or KTA crystal, or maximizing the intensity of optical parametric generation (OPG) spectra from the unseeded BBO crystal. To facilitate more reliable tuning behavior of the OPA, the WLC pulses were stretched to 1–2 picoseconds by sending the seed beam through a 5-mm thick ZnSe plate (thus the pump-seed delay was an additional parameter defining the signal/idler wavelengths). The amplified signal/idler pulses passed through a long-pass filter to suppress the 800 nm pump, and their spectra were measured with a thermoelectrically cooled InGaAs array detector (Ocean Optics NIR256-2.5). The signal/idler, as well as pump beam power values were measured with a broadband powermeter (Melles Griot, 13PEM001). The total signal + idler power values reached >3 mW for optimized KTP/KTA-OPA, and >4 mW for BBO-OPA, at peaks of respective tuning curves. The pump spectra were measured with a mini-USB spectrometer (Ocean Optics USB2000), after appropriate attenuation with VND filters.

## Additional Information

**How to cite this article**: Isaienko, O. and Robel, I. Phonon-assisted nonlinear optical processes in ultrashort-pulse pumped optical parametric amplifiers. *Sci. Rep.* **6**, 23031; doi: 10.1038/srep23031 (2016).

## Supplementary Material

Supplementary Information

## Figures and Tables

**Table 1 t1:** Fit parameters for the pump intensity dependence of the OPA outputs.

Data set	Fit to Eqn. 4	Fit to Eqn. 5
KTP-OPA, Signal/idler ~1360/2200 nm Total output power (Fig. 4b)	B = 1.25 ± 0.14 [μJ^−1/2^] C = −0.074 ± 0.011 [μJ^−1^]	B = 3.7.10^−9^ ± 4.7.10^−10^ [m/V] C = −6.3.10^−19^ ± 9.3.10^−20^ [m^2^/V^2^]
KTP-OPA, Signal/idler ~1360/2200 nm Mode [0], ~1244 nm (Fig. 4c)	B = 0.43 ± 0.04 [μJ^−1/2^] C=0 (fixed)	B = 1.3.10^−9^ ± 2.2.10^−10^ [m/V]C=0 (fixed)
KTP-OPA, Signal/idler ~1360/2200 nm Mode ν[0]–δ_1_, ~1270–1290 nm (Fig. 4c)	B = 0.502 ± 0.05 [μJ^−1/2^] C=0 (fixed)	B = 1.45.10^−9^ ± 1.3.10^−10^ [m/V] C=0 (fixed)
KTP-OPA Simulation for C=0 (Fig. 4b)	B = 1.1 [μJ^−1/2^]	
BBO-OPA Signal/idler ~1360/1920 nm (Fig. 4b)	B = 0.733 ± 0.007 [μJ^−1/2^] C=0	B = 2.15.10^−9^ ± 2.10^−12^ [m/V] C=0 (fixed)

(See Table S3 for a more extended list of fit parameters).
