# Supplementary material for: Phonon-assisted nonlinear optical processes in ultrashort-pulse pumped optical parametric amplifiers
Source: Sci Rep. 2016 Mar 15;6:23031. doi: 10.1038/srep23031 (PMC4792150; doi:10.1038/srep23031)
Supplement: Supplementary Information [file srep23031-s1.pdf]

Supporting information

Phonon-assisted nonlinear optical processes in ultrashort-pulse pumped optical parametric amplifiers

Oleksandr Isaienko and István Robel

Chemistry Division, Los Alamos National Laboratory, Los Alamos, NM 87545

1. Figure S1. Calibration verification of visible and near-IR spectrometers.

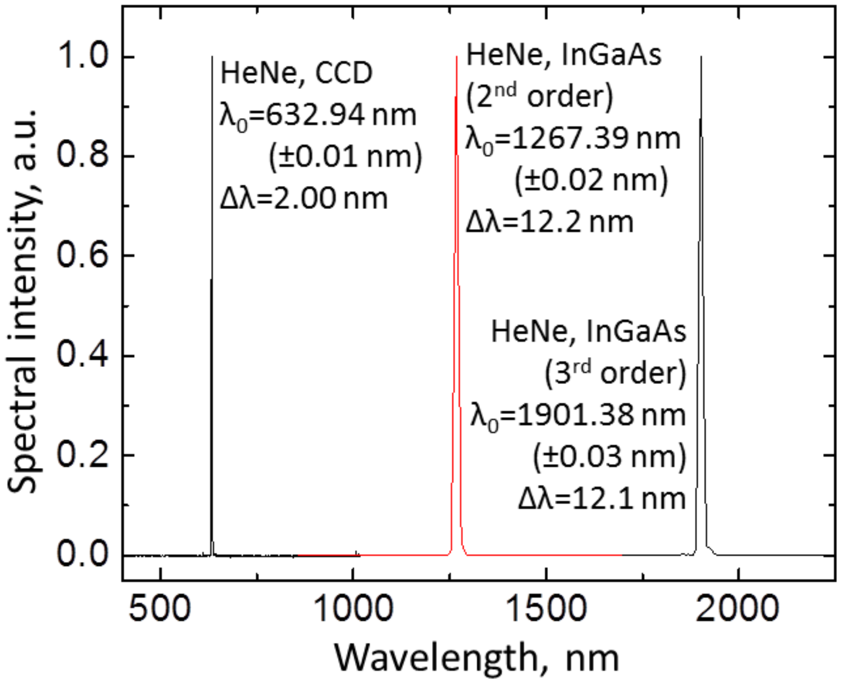

HeNe laser (632.8 nm line) spectra measured by the visible-CCD and near-IR InGaAs spectrometers. The central wavelength (λ_0) and full-width at half-maximum ($\Delta\lambda$) values are the results of Gaussian fits. Based on the resolution of the InGaAs spectrometer, we calculate the error in determination of the spectral positions of signal spectra from the OPA (and thus the redshift value Δ) to be $\approx\pm 78$ cm^{-1}, corresponding to ±2.34 THz (±9.67 meV).

2. **Figure S2. Dependence of the overall redshift Δ in KTP-OPA as a function of the pump pulse bandwidth.**

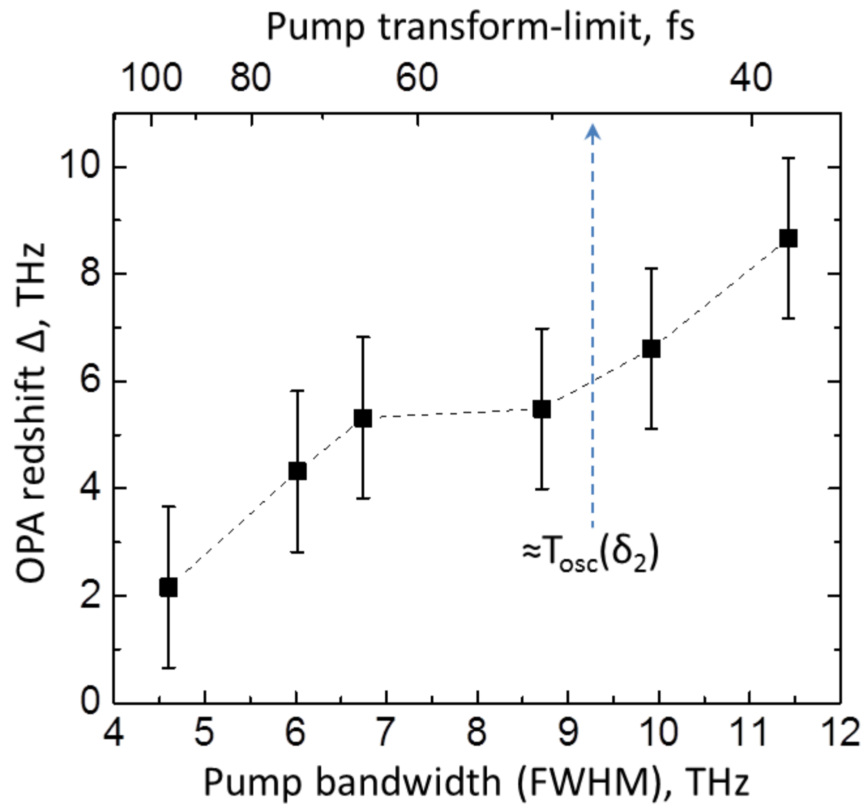

The redshift values were measured at ~25-28 μJ pump pulse energy at the crystal in all cases. The top horizontal axis displays the respective transform–limit pulse-widths (assuming Gaussian pulse shape). The vertical dashed line indicates the oscillation period of the δ_2~21 THz phonon mode. The oscillation period of the δ_2~6.9 THz phonon mode (~145 fs) is outside the range of the horizontal axes.

3. Results for pump-intensity dependence measurements, KTP-OPA, λ_s~1250 nm, λ_i~2200 nm (data in Fig. 4).

3.1. Table S1. Individual signal/idler spectra. Envelope mode spectra are shown as well (blue), as well as the expected "intrinsic signal" wavelength (dashed line).

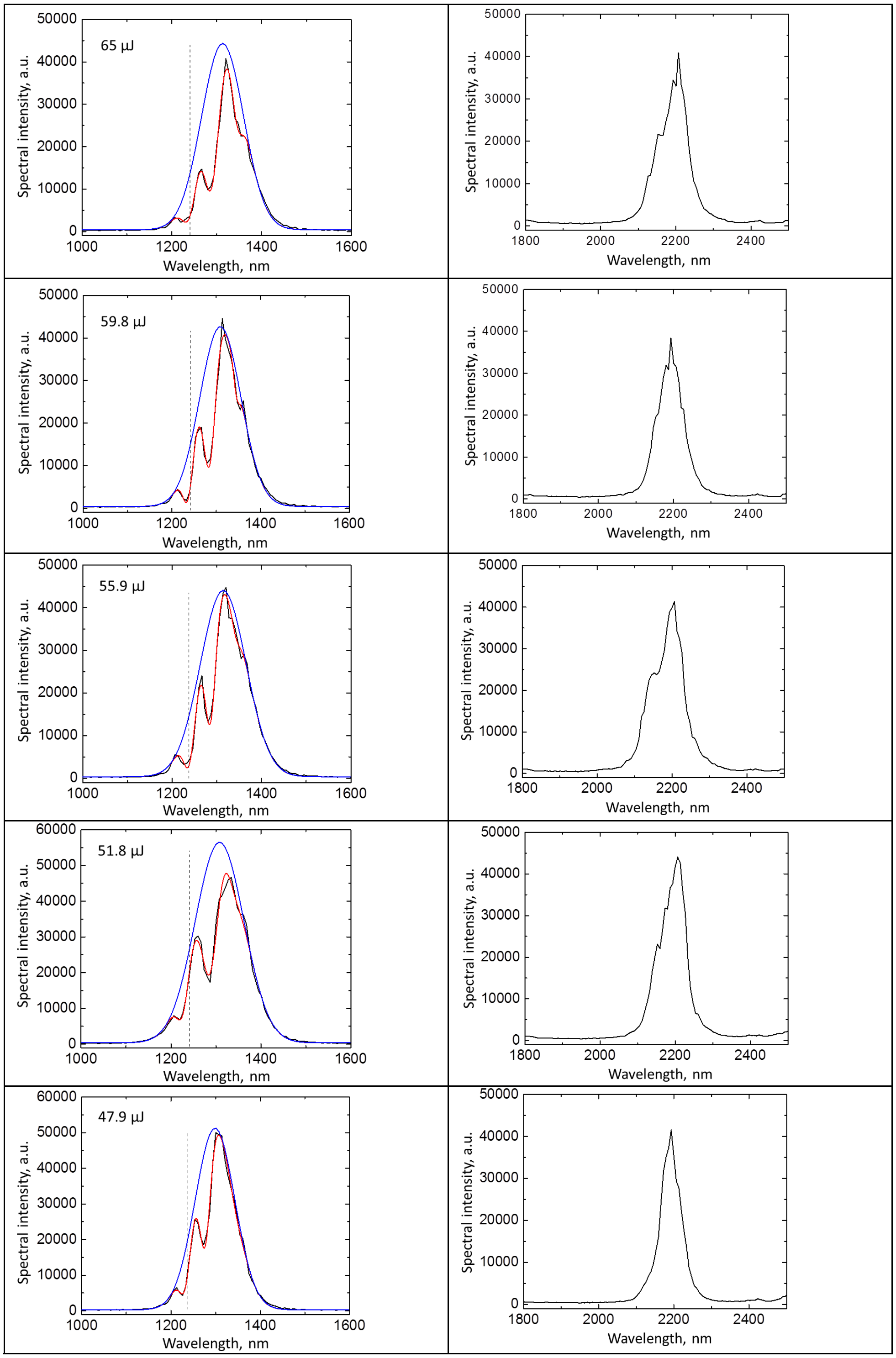

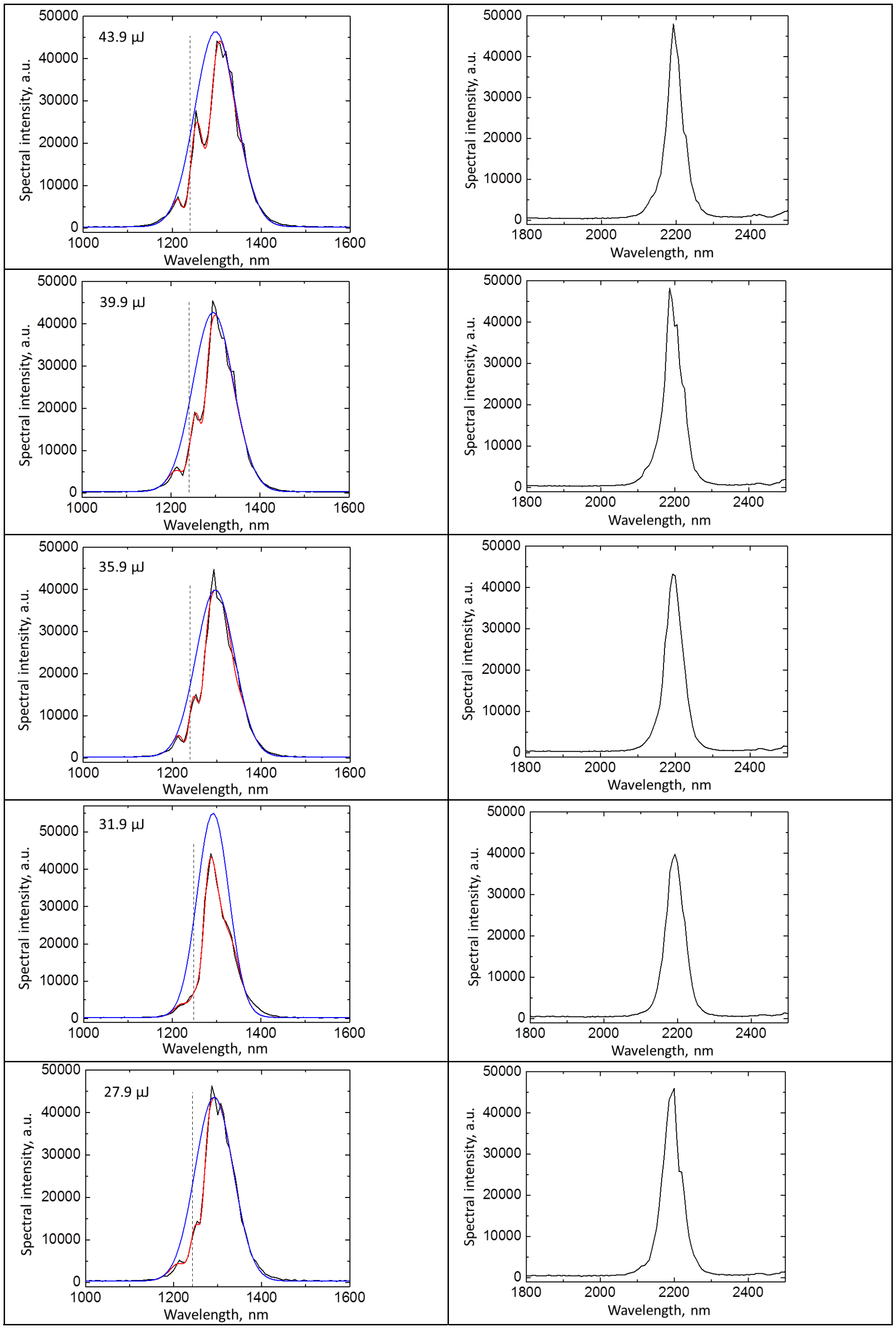
43.9 µJ
39.9 µJ
35.9 µJ
31.9 µJ
27.9 µJ
Spectral intensity, a.u.
Wavelength, nm
0
10000
20000
30000
40000
50000
1000
1200
1400
1600
1800
2000
2200
2400

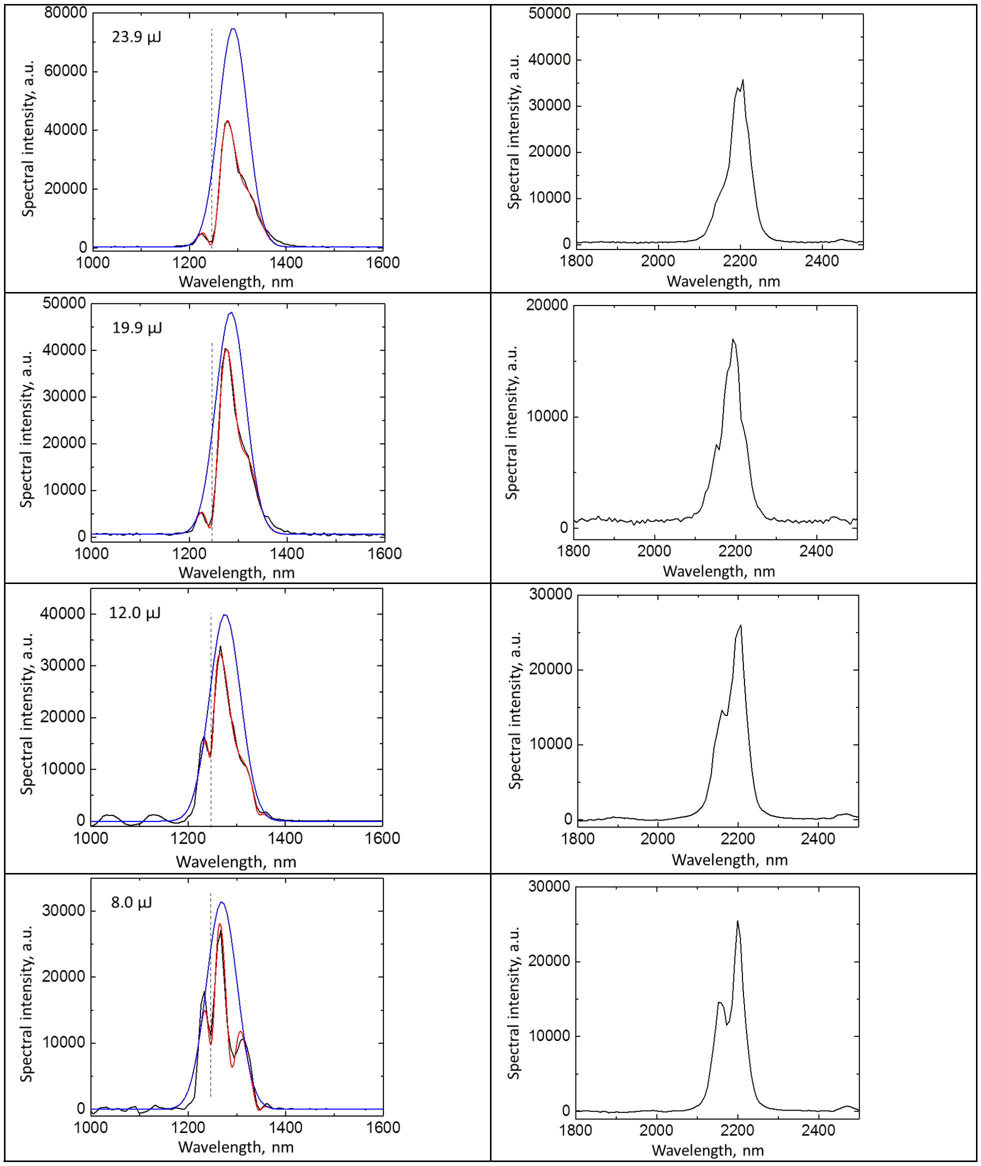
23.9 μJ
19.9 μJ
12.0 μJ
8.0 μJ
Spectral intensity, a.u.
Wavelength, nm
0
10000
20000
30000
40000
50000
60000
80000
1000
1200
1400
1600
1800
2000
2200
2400

3.2. Table S2. Compilation of multi-peak fit results for signal spectra (Table S1).

Pump energy P, μJ	A1, a.u.	λ1, nm	Δλ1, nm	A2, a.u.	λ2, nm	Δλ2, nm	A3, a.u.	λ3, nm	Δλ3, nm	A4, a.u.	λ4, nm	Δλ4, nm	Total OPA power	P1	P2	P3
65.00	43950	1313.3	66.42	-9722	1241.8	22	-28485	1289.7	23	-10139	1345.4	16	2.4	3.7110	0.2719	0.8329
59.82	42295	1309.55	65.6	-10609.8	1237.2	15.7	-27209	1285.6	18.6	-5365	1345.9	10.1	2.2	2.9809	0.1790	0.5437
55.91	43671	1314.4	72.4	-11670.8	1239.4	17.5	-24773	1286	16.3	-4869	1342.3	15	2.05	2.6128	0.1688	0.3337
51.84	56147.8	1308.1	77	-10118	1227	17.4	-32622	1287.6	24.6	-5000	1340	20.9	1.9	2.5351	0.1032	0.4706
47.85	50966	1298.8	62.7	-10401	1232	16.02	-27261	1277	16.5	-2437	1342	20	1.7	2.1469	0.1119	0.3022
43.87	46104	1297.3	66	-11008	1231.6	15.05	-22778	1277.1	17.7	-962	1343	20	1.53	1.8965	0.1033	0.2513
39.88	42451	1294.8	64.1	-10751	1236	21	-19214	1271	14.4	-500	1342	20	1.3	1.6016	0.1329	0.1629
35.89	39669	1296.8	61	-8170	1231.6	13	-15599	1263.6	14.8	-3000	1342	15	1.03	1.2231	0.0537	0.1167
31.90	54760	1292	50.65	-15704	1249	24.2	-13966	1265	14.46	-18409	1308	25.1	0.73	1.1707	0.1604	0.0852
27.91	43327	1292.3	61.1	-11255	1237.3	23.5	-15370	1263	12.48	0	1342	15	0.7	0.8458	0.0845	0.0613
23.93	74794	1290	42	-25596	1251.4	16.96	-42814	1300	28.5	0	1342	20	0.3	0.6337	0.0876	0.2462
19.94	47618	1284.6	43.2	-19245	1248.1	16	-19607	1300	21	0	1342	15	0.12	0.1846	0.0276	0.0369
12	40000	1276	45.3	-12929	1247.2	8.8	-17537	1292	25	-2500	1342.6	10	N/A			
8	31472	1268.8	44.5	-15000	1248	10	-19504	1287	15	-2000	1342	10	N/A			

Indices: “1”: envelope mode (blue lines in Table S.1); “2”: mode [0] (at intrinsic signal wavelength); “3”: mode at $\nu[0]-\delta_1$; “4”: mode at frequencies between $\nu[0]-2\delta_1$ and $\nu[0]-\delta_2$.

2.3. Figure S3. Pump energy (and pump electric field) dependence of the total OPA power (a; also shown in Fig. 4b) and the envelope mode power (b), together with fits to Eqns. 4 and 5 for corresponding values of parameter A. The corresponding values of B and C parameters are provided in Table S2.

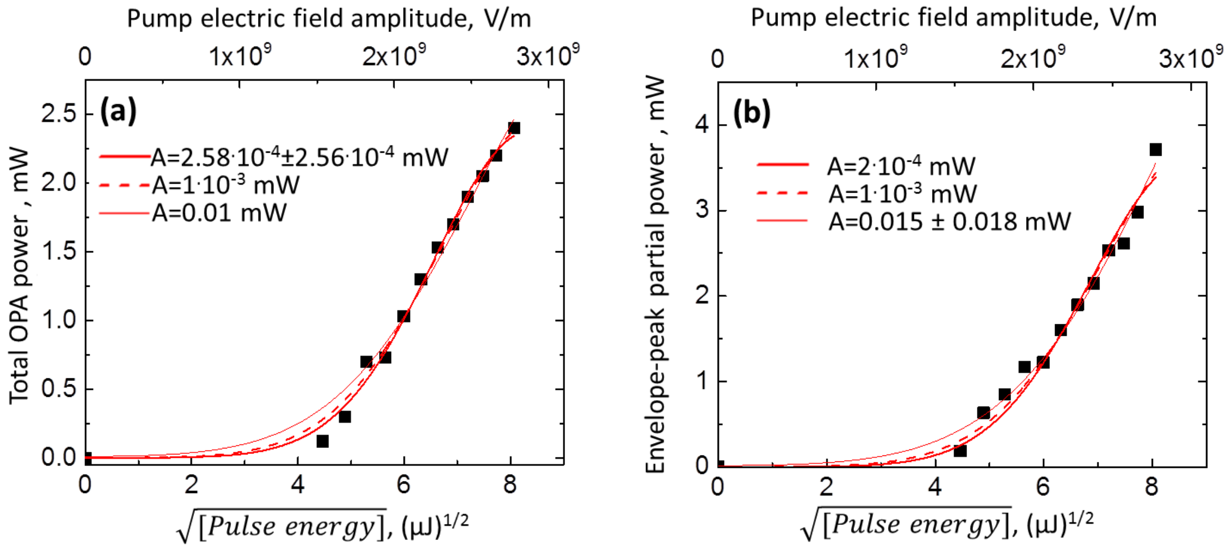

The electric field amplitudes E for the pump wave were calculated based on $I = {}^{1}/_{2}\, cn\varepsilon_0 E^2$, where n(KTP)=1.8, ε_0=8.854·10^{-12} C/(V·m), c=3·10^{8} m/s; I is the pulse peak intensity calculated based on the 50-fs pump pulsewidth and the ~300-micron diameter of the beam at the nonlinear optical crystal in the OPA.

2.4. Figure S4. Pump energy dependence of individual signal (a) and idler (b) outputs separated by their polarizations. The similar results of the fits to Eqn. 4 demonstrate that both signal and idler are generated from the same effective nonlinearity. The large errors in parameter A values are due to the fact that we artificially assign the zero output value at the zero pump power (see discussion below for Table S3).

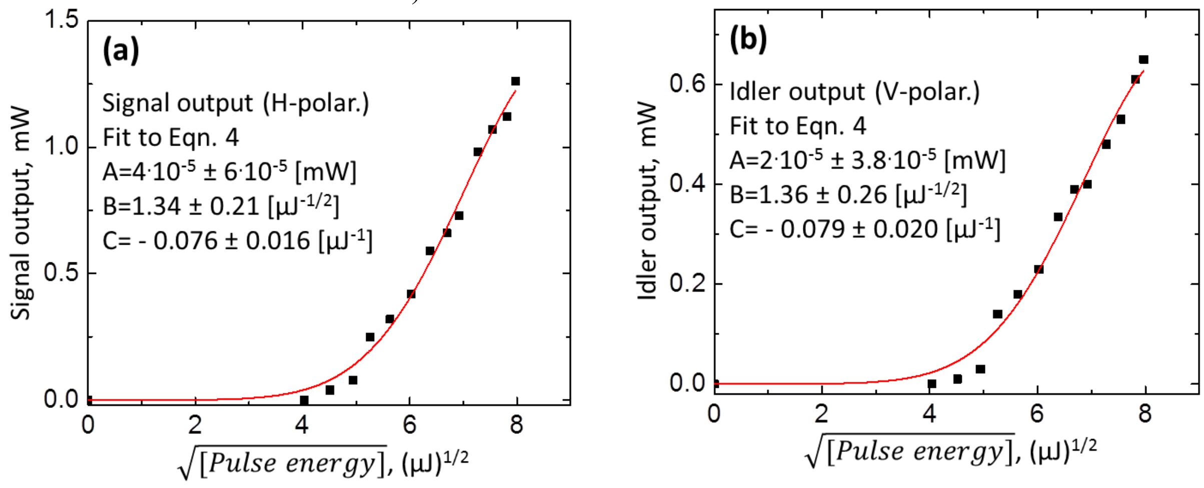

2.5. Table S3. Fit parameters of pump energy and pump electric field dependencies of the total OPA output and various modes from KTP-OPA, Fig. 4 of the main text, and Fig. S3.
The parameter is fixed to the indicated value wherever the error is not indicated.

The large error in the fit parameter A values is explained by the fact that we artificially assigned the zero power value at the zero pump intensity. However the OPA output at the zero pump power is essentially the seed power within the amplified bandwidth which may vary in the range ~10^{-4} – 10^{-3} μJ. For this reason we tested the fit results for a few values of parameter A. While the values of A change by ~two orders of magnitude, the values of the B and C parameters do not change drastically. In the main text (Fig. 4 and Table 1), we report the values of A, B and C that provide the best fit curves for each given data set.

Data set	Fit to Eqn. 4	Fit to Eqn. 5
KTP-OPA,<br>Signal/idler ~1360/2200 nm<br>Total output power<br>(Fig. 4, b; also Fig. S.3, a)	$A=2.6\cdot10^{-4} \pm 2.6\cdot10^{-4}$ [mW]<br>$B=1.25 \pm 0.14$ [μJ$^{-1/2}$]<br>$C= -0.074 \pm 0.011$ [μJ^{-1}]	$A=2.6\cdot10^{-4} \pm 2.6\cdot10^{-4}$ [mW]<br>$B=3.7\cdot10^{-9} \pm 4.7\cdot10^{-10}$ [m/V]<br>$C= -6.3\cdot10^{-19} \pm 9.3\cdot10^{-20}$ [m^2/V^2]
KTP-OPA,<br>Signal/idler ~1360/2200 nm<br>Total output power<br>(Fig. S.3, a)	$A=1\cdot10^{-3}$ [mW]<br>$B=1.06 \pm 0.011$ [μJ$^{-1/2}$]<br>$C= -0.061 \pm 0.002$ [μJ^{-1}]	$A=1\cdot10^{-3}$ [mW]<br>$B=3.07\cdot10^{-9} \pm 3.3\cdot10^{-11}$ [m/V]<br>$C= -5.1\cdot10^{-19} \pm 1.33\cdot10^{-20}$ [m^2/V^2]
KTP-OPA,<br>Signal/idler ~1360/2200 nm<br>Total output power<br>(Fig. S.3, a)	$A=1\cdot10^{-2}$ [mW]<br>$B=0.72 \pm 0.017$ [μJ$^{-1/2}$]<br>$C= -0.036 \pm 0.002$ [μJ^{-1}]	$A=1\cdot10^{-2}$ [mW]<br>$B=2.1\cdot10^{-9} \pm 4.9\cdot10^{-11}$ [m/V]<br>$C= -3.0\cdot10^{-19} \pm 2.0\cdot10^{-20}$ [m^2/V^2]
KTP-OPA,<br>Signal/idler ~1360/2200 nm<br>Mode [0], ~1244nm (Fig. 4,c)	$A=1\cdot10^{-3}$ [mW]<br>$B=0.43 \pm 0.04$ [μJ$^{-1/2}$]<br>C=0	$A=1\cdot10^{-3}$ [mW]<br>$B=1.3\cdot10^{-9} \pm 2.2\cdot10^{-10}$ [m/V]<br>C= 0
KTP-OPA,<br>Signal/idler ~1360/2200 nm<br>Mode $\nu[0] - \delta_1$, ~1270-1290nm<br>(Fig. 4, c)	$A=1\cdot10^{-3}$ [mW]<br>$B=0.502 \pm 0.05$ [μJ$^{-1/2}$]<br>C=0	$A=1\cdot10^{-3}$ [mW]<br>$B=1.45\cdot10^{-9} \pm 1.3\cdot10^{-10}$ [m/V]<br>C= 0
KTP-OPA<br>Simulation for C=0<br>(Fig. 4, c)	$A=3.1\cdot10^{-5}$ [mW]<br>B= 1.1 [μJ$^{-1/2}$]	
BBO-OPA<br>Signal/idler ~1360/1920 nm<br>(Fig. 4, c)	$A=2.58\cdot10^{-4}$ [mW]<br>(fixed to conform with KTP-OPA data)<br>$B=0.733 \pm 0.007$ [μJ$^{-1/2}$]<br>C=0	$A=2.58\cdot10^{-4}$ [mW]<br>$B=2.15\cdot10^{-9} \pm 2\cdot10^{-12}$ [m/V]<br>C= 0
KTP-OPA<br>Signal/idler ~1360/2200 nm<br>Envelope, ~1300 nm (Fig. S.3, b)	$A=2.0\cdot10^{-4}$ [mW]<br>$B=1.29 \pm 0.02$ [μJ$^{-1/2}$]<br>$C= -0.074 \pm 0.003$ [μJ^{-1}]	$A=2.0\cdot10^{-4}$ [mW]<br>$B=3.7\cdot10^{-9} \pm 6.3\cdot10^{-11}$ [m/V]<br>$C= -6.2\cdot10^{-19} \pm 2.5\cdot10^{-20}$ [m^2/V^2]
KTP-OPA<br>Signal/idler ~1360/2200 nm<br>Envelope, ~1300 nm (Fig. S.3, b)	$A=1.0\cdot10^{-3}$ [mW]<br>$B=1.06 \pm 0.018$ [μJ$^{-1/2}$]<br>$C= -0.058 \pm 0.002$ [μJ^{-1}]	$A=1.0\cdot10^{-3}$ [mW]<br>$B=3.07\cdot10^{-9} \pm 5.2\cdot10^{-11}$ [m/V]<br>$C= -4.9\cdot10^{-19} \pm 2.1\cdot10^{-20}$ [m^2/V^2]
KTP-OPA<br>Signal/idler ~1360/2200 nm<br>Envelope, ~1300 nm (Fig. S.3, b)	$A=0.015 \pm 0.018$ [mW]<br>$B=0.66 \pm 0.17$ [μJ$^{-1/2}$]<br>$C= -0.029 \pm 0.013$ [μJ^{-1}]	$A=0.015 \pm 0.018$ [mW]<br>$B=1.9\cdot10^{-9} \pm 5\cdot10^{-10}$ [m/V]<br>$C= -2.5\cdot10^{-19} \pm 1.1\cdot10^{-19}$ [m^2/V^2]

4. Results for pump-intensity dependence measurements, KTA-OPA, λ_s~1400 nm, λ_i~1900 nm.

4.1. Table S4. Individual signal/idler spectra. In selected signal spectra, the envelope mode spectrum (blue line), as well as the expected position of the "intrinsic" signal mode (dotted vertical line) are shown.

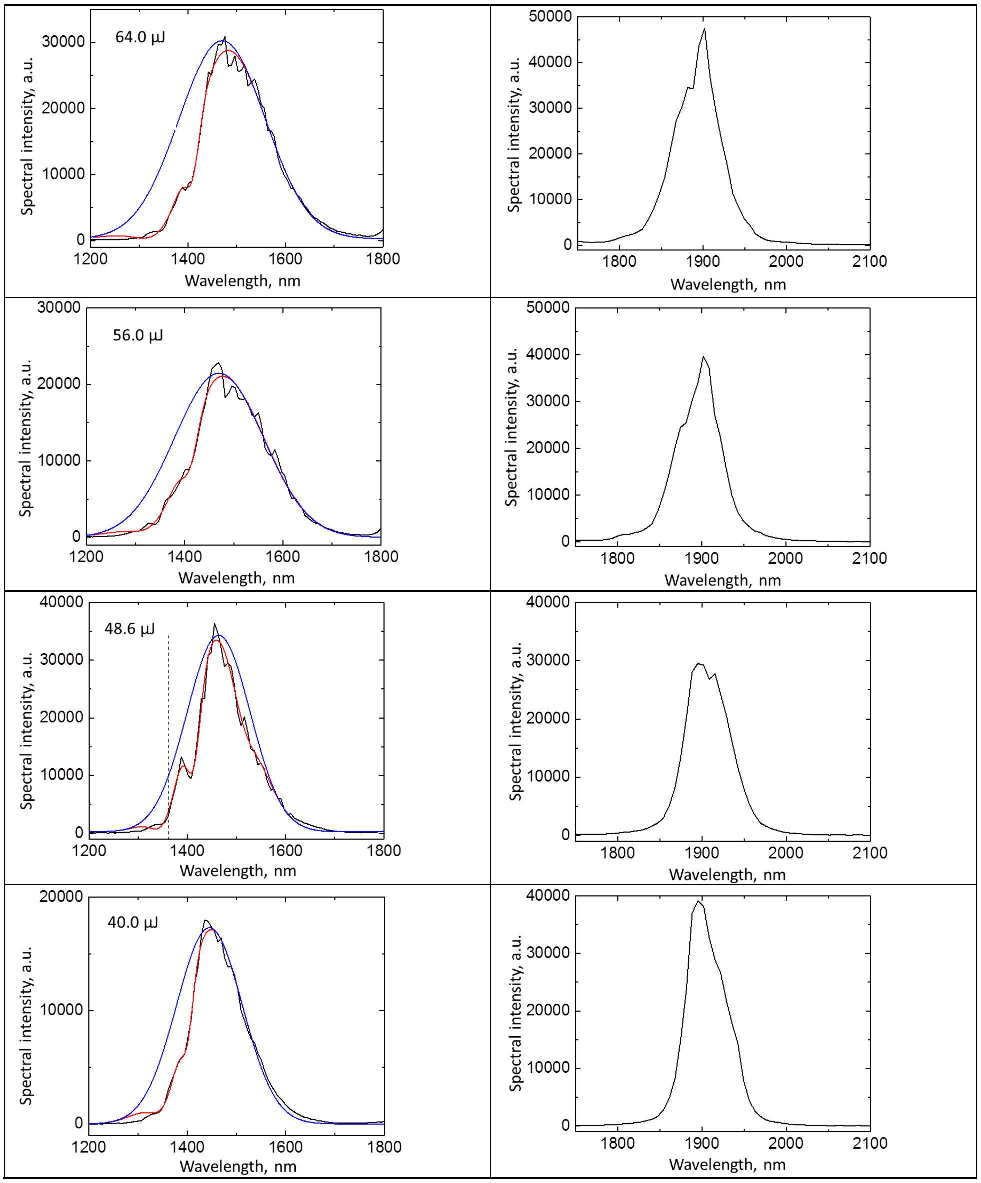

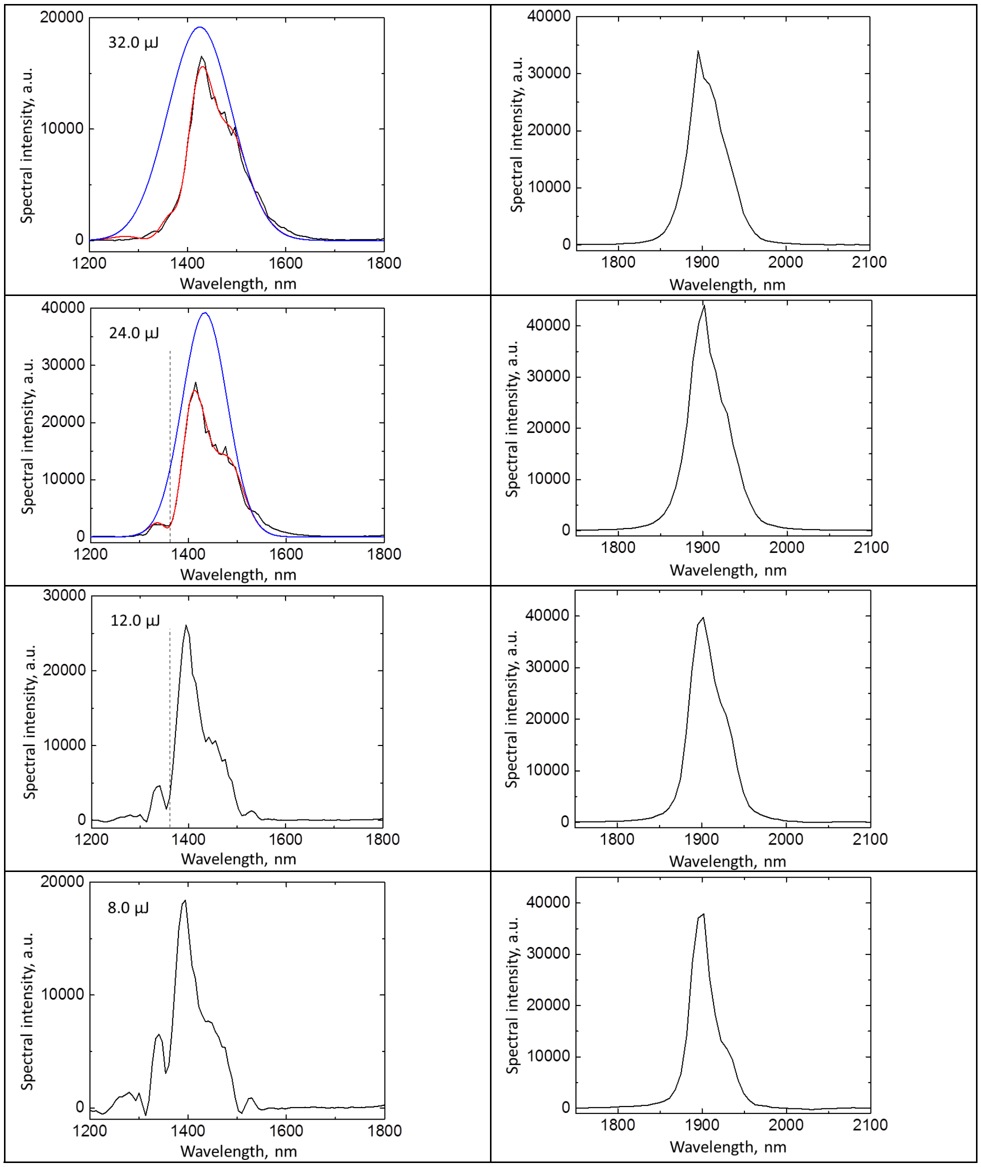
32.0 μJ
24.0 μJ
12.0 μJ
8.0 μJ
Spectral intensity, a.u.
Wavelength, nm
1200
1400
1600
1800
2000
2100
1900
0
10000
20000
30000
40000

4.2. **Table S5.** Compilation of multi-peak fit results for signal spectra (from Table S4).

Pump energy P, μJ	A1, a.u.	λ1, nm	Δλ1, nm	A2, a.u.	λ2, nm	Δλ2, nm	A3, a.u.	λ3, nm	Δλ3, nm	A4, a.u.	λ4, nm	Δλ4, nm	Total OPA power	P_1	P_2	P_3
64	30040	1470	125	-10504	1370	76	-6575	1410	20	0	N/A	N/A	2.9	3.8544	0.8194	0.1350
56	21479	1471	128	-6063	1360.3	69.5	-4642	1412	24	0	N/A	N/A	2.6	3.2250	0.4943	0.1307
48.568	34100	1464	91	-6064	1358	36	-13209	1413.7	20.5	-6708	1512	35	1.8	2.3476	0.1652	0.2049
40	17322	1445.4	93.8	-5216	1365	45	-3890	1401	16.4	0	N/A	N/A	1.3	1.5926	0.2301	0.0625
32	19181	1425.6	92	-7887	1360	58.6	-5173	1388.5	25	-4208	1462	29.2	0.8	1.3441	0.3520	0.0985
24	39265	1433.4	65	-10689	1369.8	25	-1979	1390	14	-20898	1446.5	37.4	0.2	0.3459	0.0362	0.0038

Indices: "1": envelope mode (blue lines in Table S.1); "2": mode [0] (at intrinsic signal wavelength); "3": mode at $\nu[0]-\delta_1$; "4": mode at frequencies between $\nu[0]-2\delta_1$ and $\nu[0]-\delta_2$.

4.3. Figure S5. KTA-OPA pump pulse energy (and pump electric field) dependences of the total OPA output (a), the envelope mode (b) and the sum of partial modes at frequencies ν[0] and ν[0] – δ_1 (c) together with fits to Eqns. 4 and 5. Corresponding values of the fit parameter A are indicated.

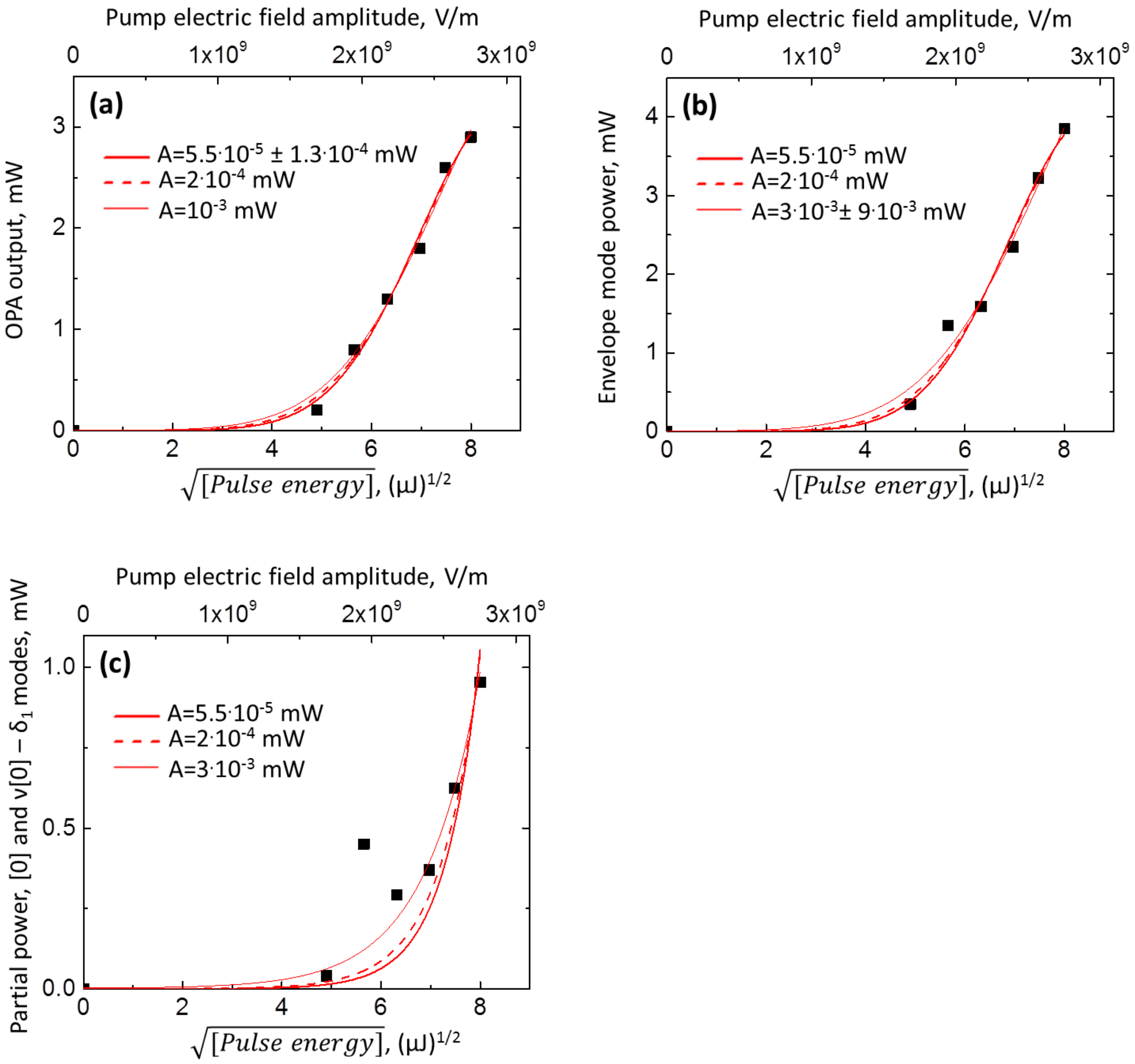

The electric field amplitudes E for the pump wave were calculated based on $I = {}^{1}/_{2}\, cn\varepsilon_0 E^2$, where n(KTA)=1.8, ε_0=8.854·10^{-12} C/(V·m), c=3·10^{8} m/s; I is the pulse peak intensity calculated based on the 50-fs pump pulsewidth and the ~300-micron diameter of the beam at the nonlinear optical crystal in the OPA.

3.4. Table S6. Fit parameters of pump energy and pump electric field dependencies of the total OPA output and various modes from KTA-OPA, Fig. S5.

The parameter is fixed to the indicated value wherever the error is not indicated.

Data set	Fit to Eqn. 4	Fit to Eqn. 5
Total output power<br>(a)	$A=5.5\cdot10^{-5} \pm 1.3\cdot10^{-4}$ [mW]<br>$B=1.42 \pm 0.33$ [$\mu J^{-1/2}$]<br>$C= -0.08 \pm 0.02$ [μJ^{-1}]	$A=5.5\cdot10^{-5} \pm 1.3\cdot10^{-4}$ [mW]<br>$B=4.1\cdot10^{-9} \pm 9.7\cdot10^{-10}$ [m/V]<br>$C= -6.9\cdot10^{-19} \pm 2.1\cdot10^{-19}$ [m^2/V^2]
Total output power<br>(a)	$A=2\cdot10^{-4}$ [mW]<br>$B=1.24 \pm 0.02$ [$\mu J^{-1/2}$]<br>$C= -0.069 \pm 0.003$ [μJ^{-1}]	$A=2\cdot10^{-4}$ [mW]<br>$B=3.6\cdot10^{-9} \pm 6.6\cdot10^{-11}$ [m/V]<br>$C= -5.8\cdot10^{-19} \pm 2.5\cdot10^{-20}$ [m^2/V^2]
Total output power<br>(a)	$A=1\cdot10^{-3}$ [mW]<br>$B=1.0 \pm 0.02$ [$\mu J^{-1/2}$]<br>$C= -0.052 \pm 0.003$ [μJ^{-1}]	$A=1\cdot10^{-2}$ [mW]<br>$B=2.9\cdot10^{-9} \pm 7\cdot10^{-11}$ [m/V]<br>$C= -4.4\cdot10^{-19} \pm 2.7\cdot10^{-20}$ [m^2/V^2]
Envelope mode power<br>(b)	$A=5\cdot10^{-5}$ [mW]<br>$B=1.48 \pm 0.03$ [$\mu J^{-1/2}$]<br>$C= -0.09 \pm 0.004$ [μJ^{-1}]	$A=5\cdot10^{-5}$ [mW]<br>$B=4.3\cdot10^{-9} \pm 9.6\cdot10^{-11}$ [m/V]<br>$C= -7.2\cdot10^{-19} \pm 3.8\cdot10^{-20}$ [m^2/V^2]
Envelope mode power<br>(b)	$A=2\cdot10^{-4}$ [mW]<br>$B=1.28 \pm 0.03$ [$\mu J^{-1/2}$]<br>$C= -0.072 \pm 0.004$ [μJ^{-1}]	$A=2\cdot10^{-4}$ [mW]<br>$B=3.7\cdot10^{-9} \pm 8.6\cdot10^{-11}$ [m/V]<br>$C= -6.0\cdot10^{-19} \pm 3.4\cdot10^{-20}$ [m^2/V^2]
Envelope mode power<br>(b)	$A=0.003\pm 0.009$ [mW]<br>$B=0.89 \pm 0.38$ [$\mu J^{-1/2}$]<br>$C= -0.044 \pm 0.03$ [μJ^{-1}]	$A=0.003\pm 0.009$ [mW]<br>$B=2.6\cdot10^{-9} \pm 1.2\cdot10^{-9}$ [m/V]<br>$C= - 3.8\cdot10^{-19} \pm 2.4\cdot10^{-19}$ [m^2/V^2]
Power of modes at frequencies $\nu[0]$ and $\nu[0] - \delta_1$<br>(“intrinsic signal”)<br>(c)	$A=5\cdot10^{-5}$ [mW]<br>$B=0.70 \pm 0.01$ [$\mu J^{-1/2}$]<br>C= 0	$A=5\cdot10^{-5}$ [mW]<br>$B=2.04\cdot10^{-9} \pm 3\cdot10^{-11}$ [m/V]<br>C= 0
Power of modes at frequencies $\nu[0]$ and $\nu[0] - \delta_1$<br>(“intrinsic signal”)<br>(c)	$A=2\cdot10^{-4}$ [mW]<br>$B=0.62 \pm 0.01$ [$\mu J^{-1/2}$]<br>C= 0	$A=2\cdot10^{-4}$ [mW]<br>$B=1.8\cdot10^{-9} \pm 2.7\cdot10^{-11}$ [m/V]<br>C= 0
Power of modes at frequencies $\nu[0]$ and $\nu[0] - \delta_1$<br>(“intrinsic signal”)<br>(c)	$A=0.003$ [mW]<br>$B=0.45 \pm 0.01$ [$\mu J^{-1/2}$]<br>C= 0	$A=0.003$ [mW]<br>$B=1.3\cdot10^{-9} \pm 2\cdot10^{-11}$ [m/V]<br>C= 0

5. Figure S6. Temporal evolution of the parametric gain in KTA crystal when pumped by ~50-fs 800-nm pulses.

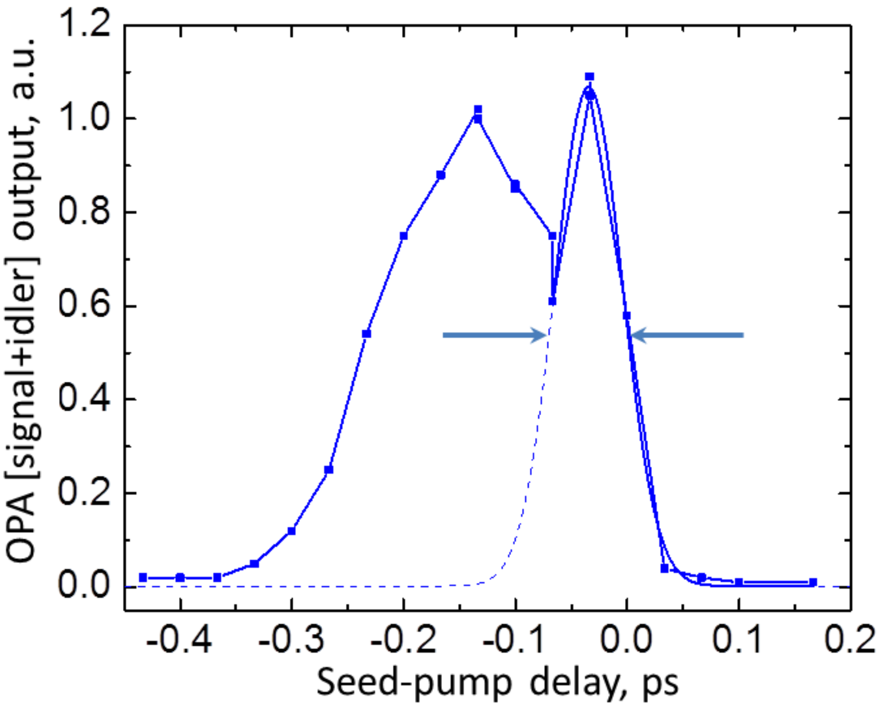

The FWHM width of the instantaneous component (fit to a Gaussian, dashed line) is ~71 fs (indicated with arrows), corresponding to ~50.4-fs pump pulsewidth as the longest estimated value. The ZnSe plate was removed from the white-light continuum seed beam path for this measurement. The KTA crystal was tuned to select the signal wavelength at ~1500 nm where the group-velocity dispersion imposed on the near-IR portion of the continuum seed pulses by routing optics is minimized. The front (trailing) edge of the pump pulse is at the positive (negative) delay values.

6. Figure S7. Spectrum of single-filament white-light continuum (WLC) seed.

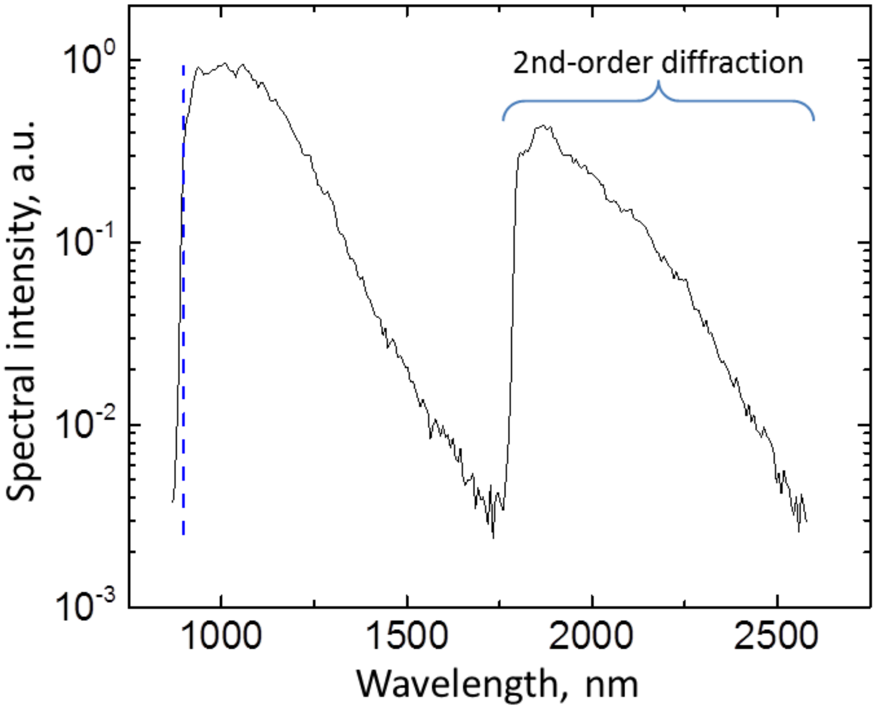

The spectrum was measured with the InGaAs array spectrometer in the absence of pump. The vertical dashed line shows the transmission edge of the 900-nm long-pass filter. The 2nd-order diffraction of the grating did not allow to measure the spectrum beyond ~1750-1800 nm, however the non-zero WLC spectral intensity at ~1750 nm indicates that the seed contained spectral components at λ>1750 nm available for optical parametric amplification (OPA). The latter was demonstrated by direct OPA of horizontally-polarized signal beyond the degeneracy point (e.g., Fig. 1a).
